# Overexpression of S100A9 in obesity impairs macrophage differentiation via TLR4-NFkB-signaling worsening inflammation and wound healing

**DOI:** 10.7150/thno.67174

**Published:** 2022-01-16

**Authors:** Sandra Franz, Anastasia Ertel, Kathrin M Engel, Jan C. Simon, Anja Saalbach

**Affiliations:** 1 Department of Dermatology, Venerology and Allergology, Faculty of Medicine, Leipzig University, Germany; 2 Institute of Medical Physics and Biophysics; Faculty of Medicine, Leipzig University, Germany

**Keywords:** S100A9, obesity, skin inflammation, wound healing, macrophage differentiation

## Abstract

**Rationale**: In obesity the fine-tuned balance of macrophage phenotypes is disturbed towards a dominance of pro-inflammatory macrophages resulting in exacerbation and persistence of inflammation and impaired tissue repair. However, the underlying mechanisms are still poorly understood.

**Methods**: Impact of obesity on macrophage differentiation was studied in high fat diet induced obese and db/db mice during skin inflammation and wound repair, respectively. Mechanisms of S100A9-mediated effects on macrophage differentiation was studied on *in vitro* generated macrophages by genomic and proteomic approaches. The role of S100A9 on macrophage differentiation was investigated by pharmacological inhibition of S100A9 during skin inflammation and wound repair in obese and db/db mice.

**Results**: We demonstrate an overexpression of S100A9 in conditions of obesity-associated disturbed macrophage differentiation in the skin. We show that saturated free fatty acids (SFA), which are increased in obesity, together with S100A9 induce TLR4 and inflammasome-dependent IL-1β release in macrophages which in turn amplifies S100A9 expression initiating a vicious cycle of sustained S100A9 overexpression in skin inflammation in obesity. We reveal a yet unrecognized impact of obesity-associated S100A9 overexpression on macrophage differentiation. S100A9 binding to TLR4 and activation of NFkB attenuates development of M2-like macrophages and induces pro-inflammatory functions in these cells. Consequently, inhibition of S100A9 restores disturbed M2-like macrophage differentiation in mouse models of obesity-associated skin inflammation and wound repair. Similarly, breaking the vicious cycle of S100A9 overexpression by dietary reduction of SFA restored M2-like macrophage activation. Improvement of skin inflammation and wound repair upon reduction of S100A9 by pharmacological inhibition or by reduction of SFA uncovers the pathogenic role of S100A9 overexpression in obesity.

**Conclusion**: This study identifies S100A9 as a previously unrecognized vital component in obesity-associated disturbed macrophage differentiation and subsequent impaired regulation of inflammation and wound repair. The findings open new opportunities for therapeutic implications for inflammatory diseases and wound repair in obesity.

## Introduction

Inflammation is a reaction of the host to infection or tissue damage with the physiological aim to restore tissue homeostasis 1. Among the many cell types acting in inflammation and tissue repair, macrophages play a central role in their orchestration by integrating specific signals from the environment into the inflammation and repair processes 2. Their spectrum of activity ranges from the induction of the initial inflammatory response, anti-microbial defense mechanisms, the phagocytosis of cell debris or apoptotic cells to the initiation of the repair of the damaged tissue and the control of the resolution of the inflammatory reaction 3. In a simplified model, macrophages are divided into macrophages with M1-like phenotypes representing one extreme - the pro-inflammatory activation state - and macrophages with M2-like phenotypes representing the opposite - anti-inflammatory and pro-repair activation states 4. Driven by danger signals from the inflammatory environment, immigrated monocytes at sites of inflammation differentiate into macrophages with pro-inflammatory M1-like phenotypes that control and drive the activation of initial immune processes. As the initial inflammatory phase subsides, composition of the macrophage population shifts towards subsets with M2-like activation profiles. The latter involve macrophage subsets that control the resolution of inflammation but also support tissue repair, e.g. by secreting growth factors to activate neighboring tissue-resident cells to realize tissue formation and vascularization 3,5. Smooth transition from M1-like to M2-like macrophage functions with several intermediate states marks a decisive step in the inflammatory resolution program 1,5. Sustained pro-inflammatory activation of macrophages and impaired induction of M2-like functions has been found in various chronic inflammatory settings including chronic wounds 6,7, lung inflammation 8, atherosclerosis 9, and insulin resistance 10. In opposite, sustained activation of macrophages with M2-like phenotypes leads to tissue fibrosis and scarring 11.

Dysbalances of M1/M2 differentiation have been observed in obesity. Numerous studies show that numbers of M1-like macrophages in adipose tissue increase in obesity inducing a pro-inflammatory microenvironment that contributes to chronic systemic low-grade inflammation 12. The activation of pro-inflammatory pathways inhibits insulin receptor signaling leading to insulin resistance and diabetes 13. It is further suggested that this dysregulation of macrophage differentiation contributes to exacerbation and prolongation of inflammation and attenuated tissue repair in obesity. Consistently, studies in several disease entities show that obesity is a risk factor for inflammatory and autoimmune disorders by increasing disease incidence and severity, and worsening disease outcomes such as in hepatic steatosis, inflammatory bowel disease, colitis, chronic kidney diseases, arteriosclerosis, and psoriasis 14-19. In addition, obesity increases the risk of adverse outcomes during acute diseases such as burns, severe trauma, and acute pancreatitis 20. Data in humans are corroborated in various animal models showing exacerbation of inflammation in sepsis-induced organ damage, psoriatic dermatitis, inflammatory bowel disease, colitis and impaired tissue repair 16-18,21-25.

Obesity is associated with hyperglycemia, dyslipidemia, hyperinsulinemia, elevation of free fatty acids (FFA), strong accumulation of adipose tissue, and increased levels of pro-inflammatory adipokines, cytokines and chemokines 26,27. However, the precise impact of these factors on the amplification and chronification of inflammation and on dysregulated macrophage differentiation in obesity is still poorly understood. Adipokines have been suggested to link obesity, adipose tissue accumulation and severity of inflammation 21,28. New emerging data indicate that metabolic components play a pivotal role in the amplification of inflammation in obesity 21,29-32. Recently, we identified a critical involvement of saturated fatty acids (SFA) in obesity-mediated exacerbation of psoriatic skin inflammation 21,28,31.

S100A9, also known as MRP14, is a Ca^2+^binding protein of the S100 family 33. It occurs as homodimer or as heterodimer with S100A8 known as calprotectin 34. Expression of S100A8 and S100A9 is massively upregulated during infection and inflammation protecting the body from the spread of pathogens. In addition to its bactericidal effects, it has been shown that S100A8 and S100A9 are particularly involved in the control of early inflammatory events through the stimulation of leukocyte recruitment and the expression of pro-inflammatory cytokines 33. Consistently, deletion of S100A9 in mice prevents initial attraction of inflammatory cells and thus inhibits induction of inflammation per se 35,36. Expression of S100A8 and S100A9 is restricted by negative feedback auto-regulatory mechanisms to prevent their overexpression 34. Nevertheless, in various inflammatory diseases as wells as in non-healing wounds expression and secretion of S100A8 and S100A9 are strongly increased. Elevated levels of S100A8 and S100A9 often correlate with the extent of the disease suggesting a pathogenic function 34,35,37. Certain S100 proteins including S100A8 and S100A9 have been associated with pathophysiological processes in obesity 38. Serum S100A8/A9 has been proposed as a marker of obesity (*38*). In our previous study, we observed an increase of S100A9 during skin inflammation in obesity 31. However, underlying mechanisms of S100A9 overexpression in obese conditions and its pathogenic role in obesity-mediated exacerbation of skin inflammation and dysregulated macrophage differentiation are still unknown.

In the present study we explore the mechanisms of obesity-associated overexpression of S100A9. We reveal a yet unrecognized impact of S100A9 on M2-like macrophage differentiation contributing to disturbed macrophage polarization and subsequent impaired resolution of skin inflammation and tissue repair in obesity.

## Results

### S100A9 disturbs the differentiation of M2-like macrophages

**Figure 1:**
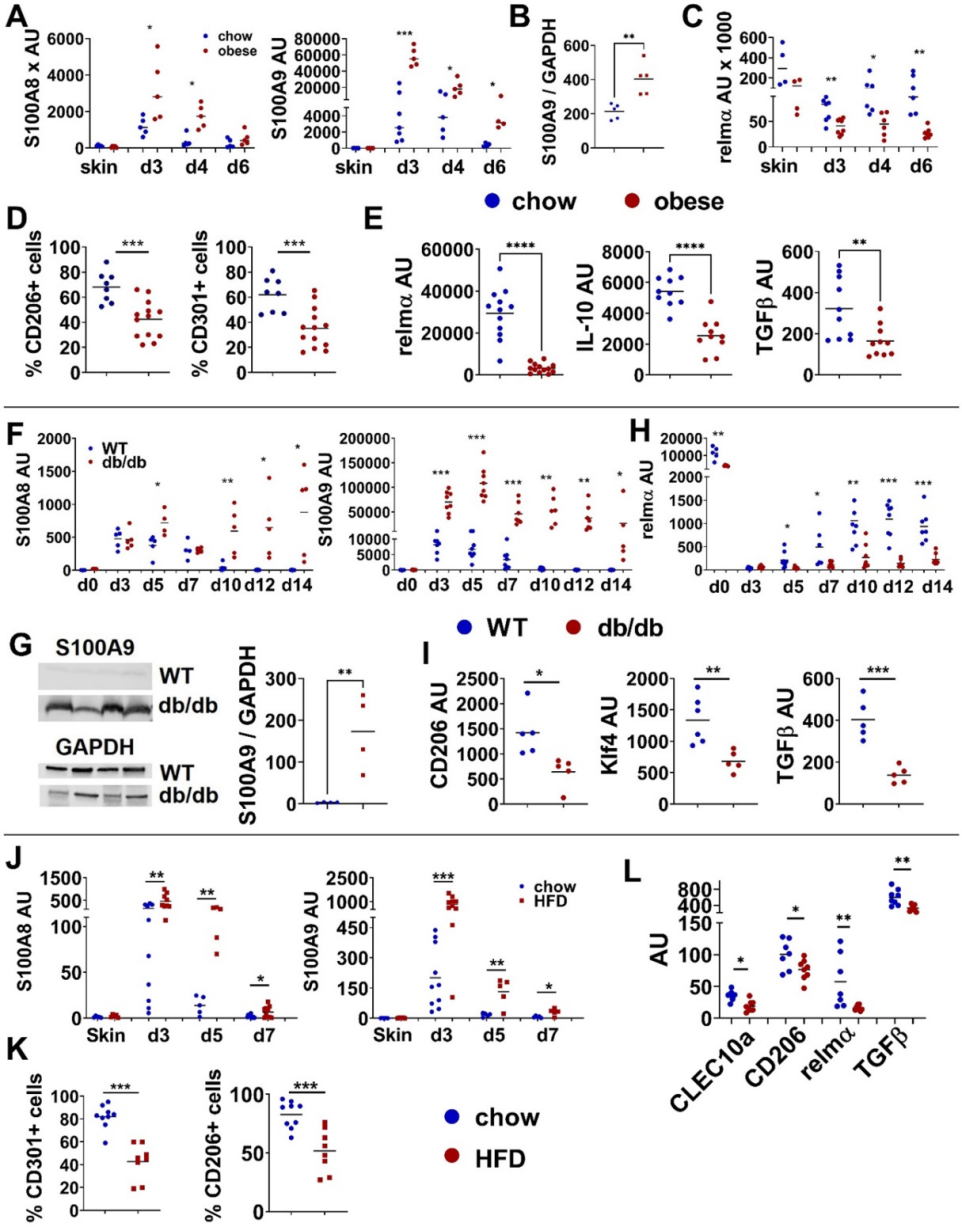
** Disturbed M2-like macrophage differentiation is associated with increased S100A9 expression. A-E)** Male C57BL/6 mice were fed with HFD for 15 weeks (obese, red) or chow diet (blue). Skin inflammation was induced by one topical application of imiquimod on the shaved back. **A)** Relative S100A8 and S100A9 gene expression in the skin over time course detected by quantitative PCR. **B)** Quantification of densitometric evaluation of S100A9 protein expression in lesional skin at day 4 by Western Blot. GAPDH was used as loading control. **C)** Relative gene expression of relmα in the skin over time course detected by quantitative PCR. **D)** Quantification of CD206+ and CD301+ expressing CD45+/CD11b+/Ly6G- cells in lesional skin (d4) by flow cytometry.** E)** Relative gene expression of M2-like markers in CD11b+ cells isolated from lesional skin at d4 analyzed by quantitative PCR. **F-I)** 13 weeks old female *db/db* mice or female C57BL/6 mice were wounded with a 6 mm punch biopsy. **F)** Relative gene expression of S100A8 and S100A9 in wound tissue analyzed by quantitative PCR analyzed at indicated time points. **G)** Detection of S100A9 in wounds at day 12 by Western Blot. GAPDH was used as loading control. Quantification of densitometric evaluation of S100A9 protein expression.** H)** Relative gene expression of relmα in wound tissue at indicated time points analyzed by quantitative PCR. **I)** Relative gene expression of indicated genes in CD11b+ cells isolated from wounds at day 9 analyzed by quantitative PCR. **J-L)** Male C57BL/6 mice were fed with HFD for 6 weeks (HFD, red) or chow diet (blue). Skin inflammation was induced by one topical application of imiquimod on the shaved back and followed over time. **J)** S100A8 and S100A9 expression in the inflamed skin detected by quantitative PCR. **K)** Quantification of CD206+ and CD301+ expressing CD45+/CD11b+/Ly6G- cells in lesional skin at day 4 by flow cytometry.** L)** Relative gene expression of M2-like markers in CD11b+ cells isolated from lesional skin analyzed at day 5 by quantitative PCR. Each symbol represents one mouse. Mean is indicated. Unpaired t-test or Mann-Whitney test: *P < 0.05, **P < 0.01, ***P < 0.001.

Since in obesity proper macrophage differentiation is disturbed, we asked for the mechanisms of obesity-associated dysregulation of macrophage differentiation and its consequences for inflammation and tissue repair in the skin. Interestingly, we observed a pathological S100A9 overexpression in conditions of disturbed macrophage differentiation. First, we induced an acute skin inflammation in high fat diet (HFD)-induced obese mice or lean control mice, to mimic the situation of an obesity-associated increased inflammatory response in the skin. Mice were fed a HFD for 15 weeks resulting in increased body weight, impaired glucose metabolism and elevated serum levels of SFA (Figure S1A). Upon application of imiquimod (IMQ) to induce an acute skin inflammation HFD-induced obese mice develop an increased and prolonged inflammatory response in the skin in comparison to lean control mice (Figure S1B). S100A8 and S100A9 are strongly induced in both lean and obese mice upon induction of inflammation (Figure 1A). Throughout the course of inflammation expression of both are significantly higher in the skin of obese mice in comparison to lean control mice. Interestingly, while S100A9 expression decreases rapidly in control mice and reaches baseline 6 days after induction of inflammation, S100A9 expression remains high in obese mice and declines much more slowly with time (Figure 1A). Western Blot analysis of skin tissue at day 4 after IMQ treatment confirms the increased expression of S100A9 protein in lesional skin of obese mice in comparison to lean controls (Figure 1B). At the same time, obese mice show an impaired regulation of the M2-like macrophage marker, relmα, in the course of skin inflammation. In early inflammation (day3) relmα expression is reduced in both lean and obese mice, but much stronger in obese mice (Figure 1C). In lean mice expression of relmα increases again beginning with day 4, the same time point when S100A9 expression starts to decline. In contrast, in obese mice relmα expression is not induced and sustains at low level until the end of observation period, day 6 after induction of inflammation (Figure 1C). The data suggest an impairment of M2 like macrophage development during skin inflammation in obese mice. Indeed, levels of CD206 or CD301 positive cells in the population of CD11b+ myeloid cells isolated from inflamed skin are reduced in obese mice as detected by flow cytometry (Figure 1D). Analysis of gene expression of M2 like macrophage markers in myeloid cells isolated from lesional skin by magnetic cell separation confirms the dysregulation of M2 like macrophages during inflammation in obese mice. Expression of typical M2-like macrophage markers like relmα, TGFβ and IL-10 are decreased in myeloid cells isolated from lesional skin 4 days after induction of inflammation compared to lean control mice (Figure 1E).

Since M1/M2 transition is a crucial step during tissue repair we compared S100A8 and S100A9 during skin wound healing in wildtype mice and in *db/db* mice, the latter representing an established model of obesity-associated impaired wound healing with disturbed macrophage differentiation 7. Of note, we observed a similar dysregulation of S100A8 and S100A9 and M2-like macrophage markers as in the obesity-associated skin inflammation model. In both, wildtype and *db/db* mice, S100A8 and S100A9 expression is rapidly induced upon injury (Figure 1F). Specifically, S100A9 showed a significantly higher expression level in *db/db* mice compared to wildtype mice (Figure 1F). Western Blot analysis proves the increased expression of S100A9 in wounds of *db/db* compared to wildtype mice at day 12 post-wounding (Figure 1G). Importantly, expression of S100A9 declines in wildtype mice up to day 7 after wounding while in *db/db* mice it persists until late time points (Figure 1F). Decrease of S100A9 in wounds of wildtype mice coincides with the induction of the expression of the M2-marker relmα (Figure 1H). In contrast, in wounds of *db/db* mice relmα induction is strongly impaired (Figure 1H). Reduced gene expression of CD206, CD163 and Klf4 in CD11b+ myeloid cells isolated from the wounds at day 9 substantiates a disturbed M2-like macrophage differentiation in delayed-healing wounds in *db/db* mice (Figure 1I) when increased levels of S100A8 and S100A9 still persist.

We recently identified SFA as one key player in obesity-mediated exacerbation of skin inflammation 31. We were therefore wondering whether SFA are involved in increased S100A8 and S100A9 expression and disturbed M2-like macrophages activation. We used our previously established model of short- term HFD-fed mice to study both events in the course of SFA-mediated increased skin inflammation. Mice were fed a HFD enriched in SFA for only 6 weeks while control mice received normal chow diet. Short-HFD mice had a normal body weight and healthy glucose metabolism but showed elevated serum levels of SFA (Figure S1C). After application of IMQ short-HFD mice present a prolonged inflammation with increased severity, reflected by increased erythema and scaling (Figure S1D). As observed in the skin inflammation model in obese mice and the *db/db* wound healing model, S100A8 and S100A9 expression in the inflamed skin of short-HFD is increased in the course of inflammation in comparison to chow-fed control mice (Figure 1J). In parallel, levels of CD206 or CD301 positive cells in the population of CD11b+ myeloid cells are reduced in short-HFD mice detected by flow cytometry (Figure 1K). Down-regulation of M2-like macrophage gene expression markers, including CD206, CD301, relmα, and TGFβ in the skin myeloid cells of short-HFD proved the disturbed M2-like macrophage activation in these mice (Figure 1L).

Since all three models of obesity/HFD-associated increased inflammation in the skin present defective M2-like macrophage induction that coincides with the overexpression of S100A8 and S100A9 in the inflamed skin tissue, and since macrophages express receptors for both such as TLR4 and RAGE, we asked whether S100A8 and S100A9 impair M2-like macrophage differentiation. First, we excluded direct effects of hyperglycemia and hyperlipidemia on M2 macrophage differentiation. Peritoneal macrophages were differentiated into M2-like macrophages by addition of IL-4. As shown in Figure S2 both palmitic acid (PA) and high glucose (25 mM) have no effect on M2-differentiation as demonstrated by the unaltered gene expression of typical M2-like macrophage markers such as relmα, CD206, CLEC10a (CD301).

**Figure 2:**
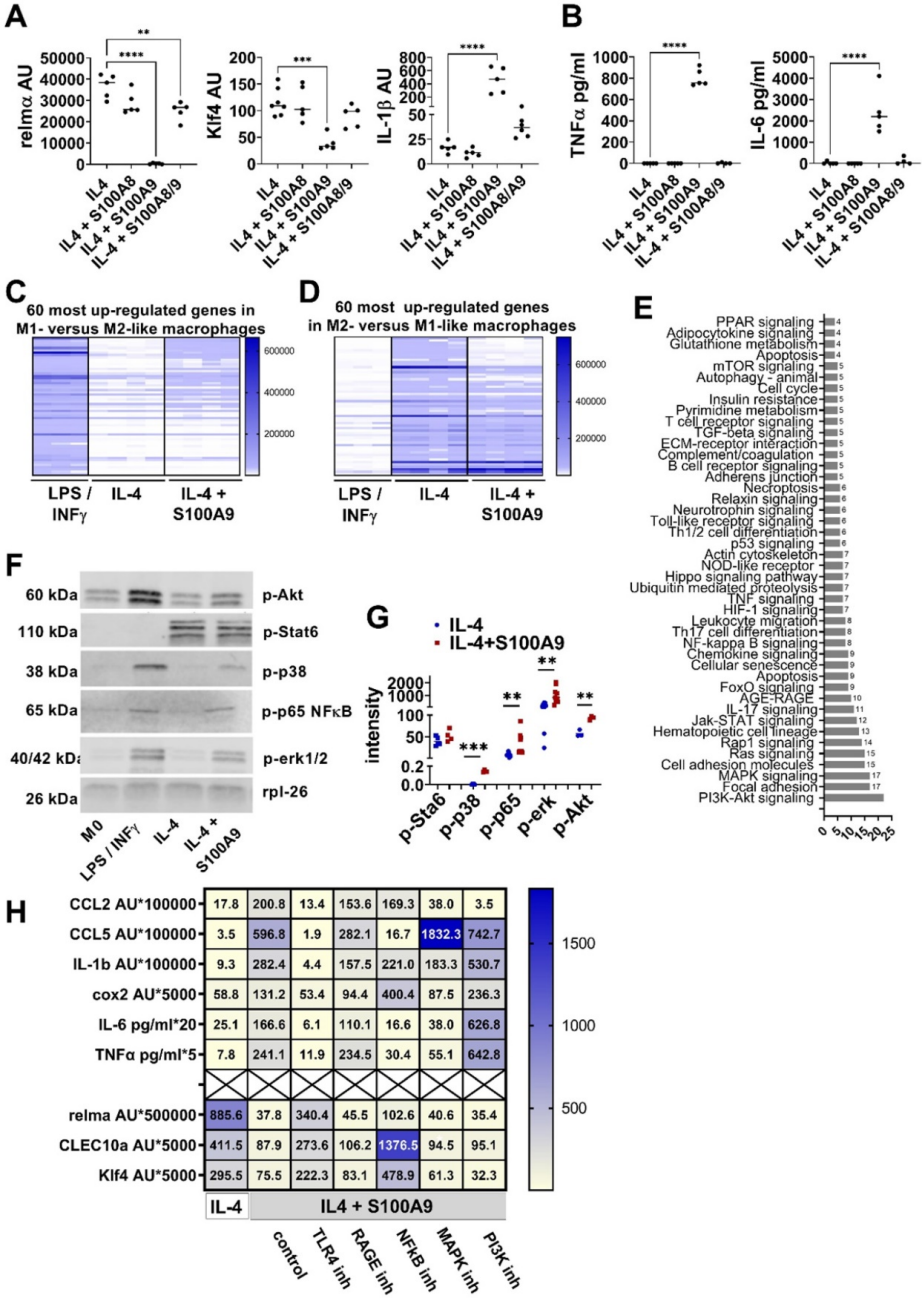
** S100A9 impairs M2-like macrophage differentiation. A/B)** Peritoneal cells were incubated with IL-4 or IL-4+S100A8 homodimer, IL4+S100A9 homodimer or IL-4+S1008/A9 heterodimer. **A)** Gene expression analysis by quantitative PCR. **B)** Cytokine levels in the supernatant detected by ELISA. Each symbol represents macrophages from one mouse. Mean is indicated. ANOVA with multiple comparisons *P < 0.05, **P < 0.01, ***P < 0.001, **** P < 0.0001. **C/D)** Peritoneal macrophages were incubated with LPS / INFγ or IL-4 to induce a M1 and M2-like macrophage phenotype, respectively. S100A9 was added during M2-like macrophage differentiation. Genome wide expression analysis. Display of the 60 most upregulated gene in **(C)** M1- versus M2-like macrophages and **(D)** M2- versus M1-like macrophages. **E)** Pathway analysis using the KEGG database of differentially expressed proteins and phosphoproteins with a log fold change of > 2 or < 0.5 identified by proteome and phospho-proteome microarray. Macrophages differentiated with IL-4 and IL-4+S100A9 of two different mice were compared. **F/G)** Detection and quantification of Stat6, p38, p65NFkB, erk1/2, and Akt, phosphorylation in peritoneal macrophages incubated with LPS / INFγ, IL-4 or IL-4 and S100A9 by Western Blot. Rpl-26 served as loading control. Representative Western Blots are shown **(F)**. Quantification of phosphorylation **(G)**. Each symbol represents macrophages from one mouse. Mean is indicated. Unpaired t-test or Mann-Whitney test between IL-4 and IL-4+S100A9 treated cells: *P < 0.05, **P < 0.01, ***P < 0.001, ns - not significant. **H)** Peritoneal macrophages were incubated with IL-4 or IL-4+S100A9 in the presence or absence of TLR4 signaling inhibitor (CLI-095, 1 µM), RAGE antagonistic peptide (ELKVLMEKEL, 10 µM), NFkB inhibitor (BMS-345541, 4 µM), PI3K inhibitor (Apitolisib, 5 µM), or MAPK inhibitor (Cobimitinib, 10 nM). Data represent the mean of ≥ 4 independent experiments. Numbers indicate the mean of AU (RS36-normalized) detected by quantitative PCR and protein detected by ELISA.

To study the impact of S100A8 and S100A9 on macrophage differentiation peritoneal cells from wildtype mice were differentiated *in vitro* into M2-like macrophages in the presence of S100A8 and S100A9. Since S100A9 can be found as homodimer and as heterodimer with S100A8, we compared the impact of S100A8 and S100A9 homodimer and S100A8/A9 heterodimer on M2-like macrophage differentiation. As shown in Figure 2A, only S100A9 significantly inhibits the expression of typical M2-like macrophage markers such as relmα and Klf4 while S100A8 homodimer and S100A8/A9 heterodimer has no or only a weak impact. In addition, S100A9 homodimer induces a pro-inflammatory activation state reflected by increased expression of IL-1β and release of TNFα and IL-6 while S100A8 homodimer and S100A/A9 heterodimer do not (Figure 2A-B). To better understand the effects of S100A9 on M2-like macrophage differentiation the gene expression pattern of M2-like macrophages differentiated in the presence or absence of S100A9 were compared by a genome-wide expression analysis. Peritoneal macrophages differentiated to M1-like macrophages by INFγ/LPS served as control. Comparison of gene expression in the different macrophages by a genome-wide expression analysis proves an impaired M2-like gene signature with downregulation of M2-like genes and up-regulation of M1-like genes in M2 macrophages differentiated in the presence of S100A9 (Figure 2C-D). Phenotyping of M2-like macrophage differentiation in the presence of S100A9 confirmed downregulation of typical M2-like macrophage markers such as relmα, CD163, CD206, Klf4 and CLEC10a (Figure S3A-B) and up-regulation of typical pro-inflammatory M1-associated genes including CD38, CCL2, CCL5 and Cox2 (Figure S3C).

To unravel the mechanism of S100A9-mediated control of macrophage activation, the proteome and phosphoproteome were screened on a phosphoproteomics platform including the analysis of about 1300 proteins. As shown in Figure 2E, S100A9 interferes with numerous pathways during M2-like macrophage differentiation including NFκB, mitogen-activated protein kinase (MAPK) and the Akt pathway. Phosphorylation of NFκB, erk, p38 and Akt by S100A9 detected by Western Blot suggests the involvement of these pathways in the S100A9-mediated dysregulation of M2-like macrophage differentiation (Figure 2F-G). Interestingly, S100A9 did not affect Stat6 pathway, one of the master regulators of M2-like macrophage differentiation (Figure 2F-G). To demonstrate the involvement of these pathways in the S100A9-induced dysregulation of M2-like macrophages, peritoneal cells were differentiated to M2-like macrophages by IL-4 and S100A9 in the presence of inhibitors of the NFkB, Akt and MAPK pathway. Inhibition of the NFkB pathway by BMS-345541 completely reverses S100A9-mediated inhibition of M2-like macrophage markers (relmα, CLEC10a, Klf4) and prevents the activation of almost all pro-inflammatory factors (CCL5, cox2, IL-6, TNFα) indicating the central role of NFkB in the S100A9-mediated impairment of M2-like macrophage differentiation (Figure 2H, Figure S4). In contrast, inhibition of the MAPK pathway by Cobimitinib or of the PI3K pathway by Apitolisib does not interfere with the effect of S100A9 on the expression of M2-like macrophage markers and blocked only the induction of same pro-inflammatory markers by S100A9 (Figure 2H, Figure S4).

Since TLR4 and RAGE are important recognition receptors of S100A9, TLR4 inhibitor CLI-095 and RAGE antagonistic peptide were added to unravel the contribution of these receptors. As shown in Figure 2H, blocking TLR4 recognition diminish S100A9-mediated stimulation of pro-inflammatory mediators and reverse the inhibition of M2-like marker expression while blocking RAGE does not affect S100A9-mediated changes (Figure 2H, Figure S4). Taken together, S100A9, but not S100A8, controls M2-like macrophage differentiation via the TLR4-NFkB axis.

### Pathological overexpression of S100A9 delays resolution of skin inflammation

**Figure 3:**
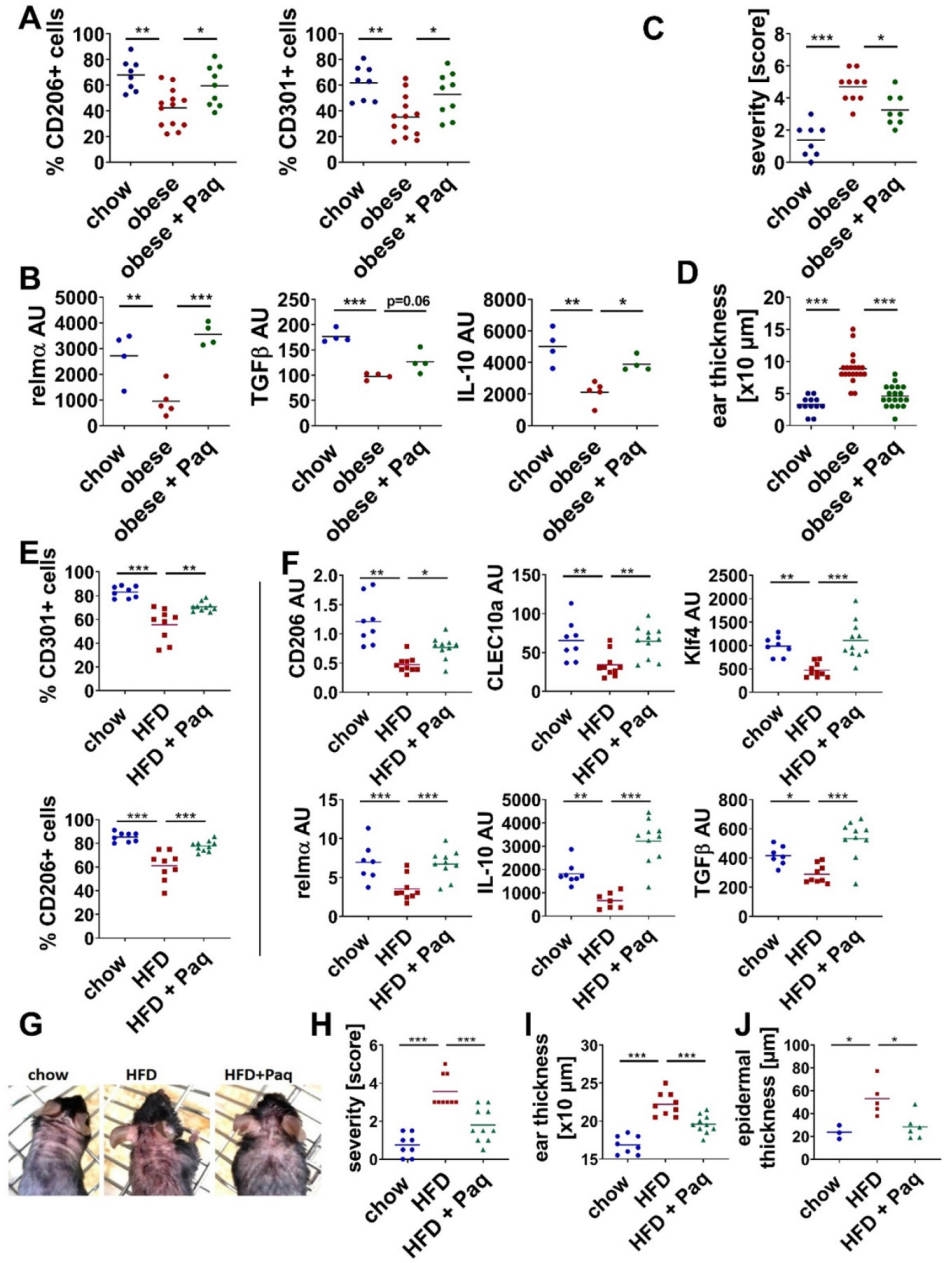
** Inhibition of S100A9 restores M2-like macrophage differentiation and prevents high fat diet- and obesity induced exacerbation of skin inflammation.** Male C57BL/6 mice were fed with chow diet (blue) or high fat diet (red) for 15 weeks (obese, **A-D**) or 6 weeks (HFD, **E-J**). Skin inflammation was induced by one topical application of imiquimod on the shaved back and ears. Paquinimod was applied 10 h after imiquimod (HFD+Paq; obese+Paq; green). Skin inflammation was analyzed at day 4. **A/E**) Quantification of CD206+ and CD301+ macrophages within CD45+/CD11b+/Ly6G- cells in the lesional skin by flow cytometry. **B/F**) Relative expression of indicated genes detected by quantitative PCR in CD11b+ myeloid cells isolated from lesional skin. **C/H**) Severity score, **D/I**) Ear swelling, **G**) Macroscopic appearance of lesional skin area. Images of mice (one representative example of each group with at least 8 mice). **J**) Epidermal thickness within skin lesions quantified in hematoxylin and eosin stainings of skin tissue sections. Each dot represents one mouse. Mean is indicated. ANOVA with multiple comparisons *P < 0.05, **P < 0.01, ***P < 0.001.

To control whether sustained overexpression of S100A9 in skin inflammation impairs M2-like macrophage differentiation and subsequent resolution of inflammation *in vivo* we prevented the activity of S100A9 by inhibition with paquinimod. Mice were fed for 6 or 15 weeks with HFD resulting in lean mice with elevated SFA (short-HFD mice) and in obese mice, respectively (Figure S1). Both mice are characterized by S100A9 overexpression upon induction of skin inflammation (Figure 1A and J). To block the activity of S100A9, mice were treated with paquinimod via the drinking water. Paquinimod belongs to a group of Quinoline-3-carboxamides (Q compounds) and inhibits the binding of S100A9 to its receptors TLR4 and RAGE 40. Since it is known that S100A9 contributes to the onset of inflammation via its chemotactic and pro-inflammatory activity 34, administration of paquinimod was started 10 h after topical IMQ application to prevent the interference with its chemotactic action at the initial phase of inflammation. As shown in Figure S5 infiltration of inflammatory cells, ear swelling and S100A9 expression are not affected at day 1 indicating that treatment with paquinimod 10 h after application of IMQ does not interfere with the initial inflammatory response and recruitment of inflammatory cells. Next, the inflammatory immune cell response was analyzed at day 4, when obese mice and short-HFD mice present increased inflammation with elevated expression of S100A9 and disturbed M2-macrophage activation (Figure 1A-E and J-L). Blocking S100A9 restores M2-like macrophage differentiation in both obese mice and short-HFD mice. In detail, the percentage of CD206+ and of CD301+ cells within the myeloid cell fraction of inflamed skin are significantly increased in obese mice after inhibition of S100A9 (Figure 3A). Analysis of myeloid cells isolated from the lesional skin confirms the increased activation of M2-like macrophages in the skin after blocking S100A9. Gene expression of relmα and functional mediators of M2-like macrophages such as TGFβ and IL-10 are restored (Figure 3B). In parallel, obesity-induced amplification of inflammation severity (Figure 3C) and ear thickening (Figure 3D) are decreased in the paquinimod-treated mice. Similar results - restored M2-like macrophage activation (Figure 3E-F) and less severe skin inflammation (Figure 3G-J) - are obtained in short-HFD mice that were treated with paquinimod.

Taken together, consistent to our *in vitro* results from Figure 2, which demonstrate the interference of M2-macrophage differentiation by S100A9, blocking of S100A9 activity restores M2-like macrophage activation during skin inflammation in obese and short-HFD mice. In parallel, blocking S100A9 normalize skin inflammation in these mice underlining a pathogenic role of S100A9 overexpression in the obesity-associated amplification of skin inflammation.

### Pathological overexpression of S100A9 attenuates wound repair

**Figure 4:**
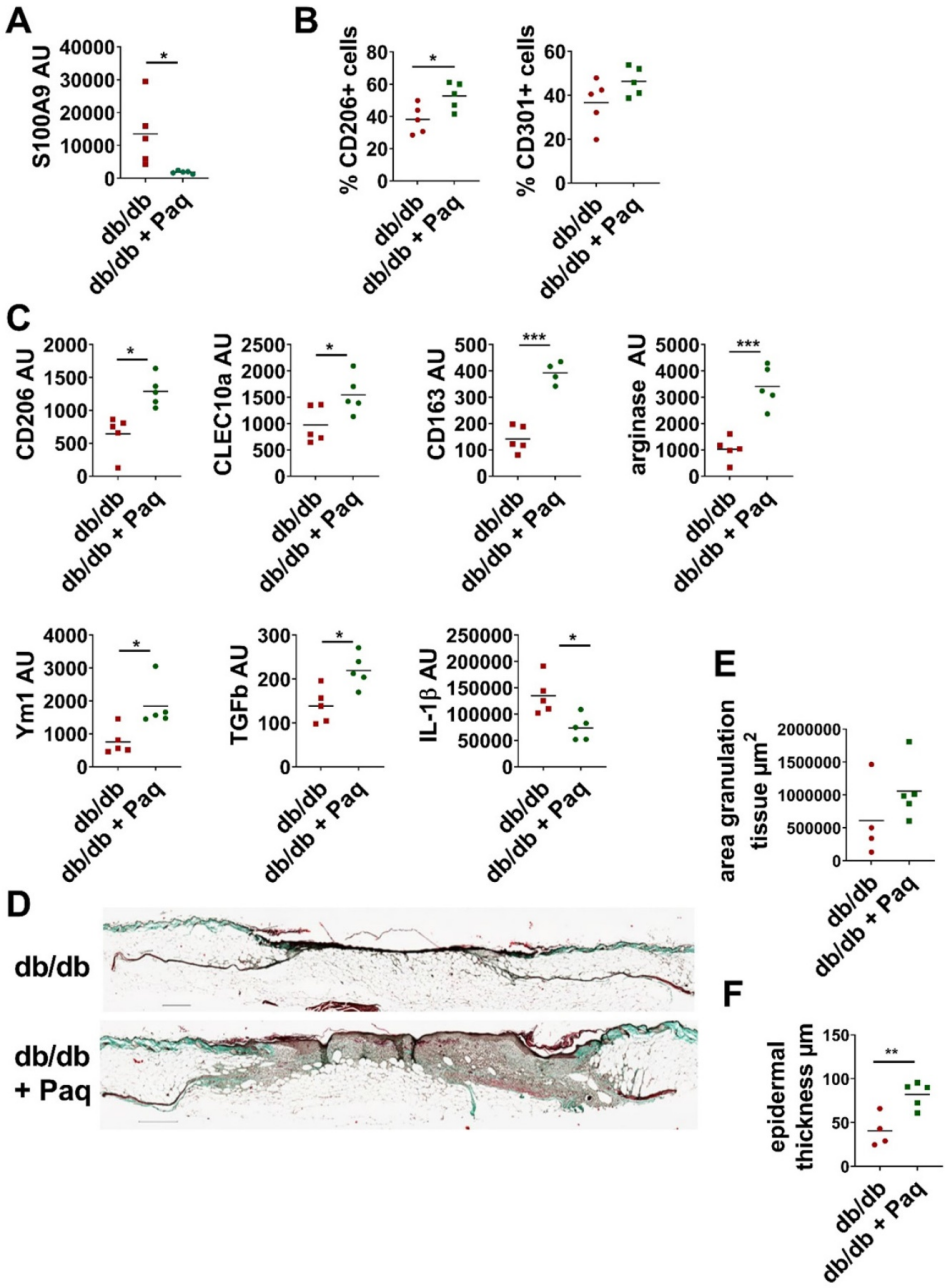
** Inhibition of S100A9 restores M2-like macrophage differentiation and improves wound healing in *db/db* mice.** *Db/db* mice were wounded with 6 mm punch biopsies. Paquinimod was applied 2 d after wounding (*db/db*+Paq; green). Wounds were analyzed 9 days after wounding. **A)** Relative expression of S100A9 in wounds detected by quantitative PCR. **B)** Quantification of CD206+ and CD301+ macrophages within CD45+/CD11b+/Ly6G- cells in the wounds by flow cytometry. **C)** Relative expression of indicated genes detected by quantitative PCR in CD11b+ myeloid cells isolated from wounds. **D)** Masson's trichrome staining of tissue sections of wounds. One representative example out of 5 *db/db* and 5 paquinimod treated *db/db* mice is shown. **E/F)** Area of granulation tissue and epidermal thickness was quantified in Masson trichrome stainings of tissue sections. Each dot represents one mouse. Mean is indicated. Unpaired t-test or Mann-Whitney test: *P < 0.05, **P < 0.01, ***P < 0.001.

Next, we investigated the link between impaired M1 to M2 transition and pathological S100A9 overexpression in obesity-mediated impaired tissue repair using the *db/db* mouse model of delayed wound healing. Full thickness excisional wounds were inflicted on the back of *db/db* mice. Two days after wounding when an initial inflammatory response had established S100A9 activity was blocked by administration of paquinimod via drinking water. Application of paquinimod reduces S100A9 expression in the wounds (Figure 4A). Consequently, confirming the inflammation model, blocking of S100A9 restores M2-like macrophage differentiation (Figure 4B-C). The percentage of CD206+ and of CD301+ cells within the myeloid cell fraction is increased in paquinimod-treated mice compared to untreated mice (Figure 4B). Analysis of CD11b+ cells isolated from the wounds confirms the restoration of M2-like macrophage differentiation upon blocking of S100A9. Gene expression of CLEC10a, CD206, CD163, arginase and Ym1 are up-regulated in the paquinimod-treated *db/db* mice (Figure 4C). In parallel, gene expression of IL-1β is decreased and expression of functional mediators of M2-like macrophages such as TGFβ is increased (Figure 4C). In parallel, wound repair is improved in the paquinimod-treated mice. Blocking S100A9 promote re-epithelization as seen by increased epidermal thickness and enhanced granulation tissue formation (Figure 4D-F).

### Mechanisms of S100A9 overexpression during inflammation and wound healing in obesity

**Figure 5:**
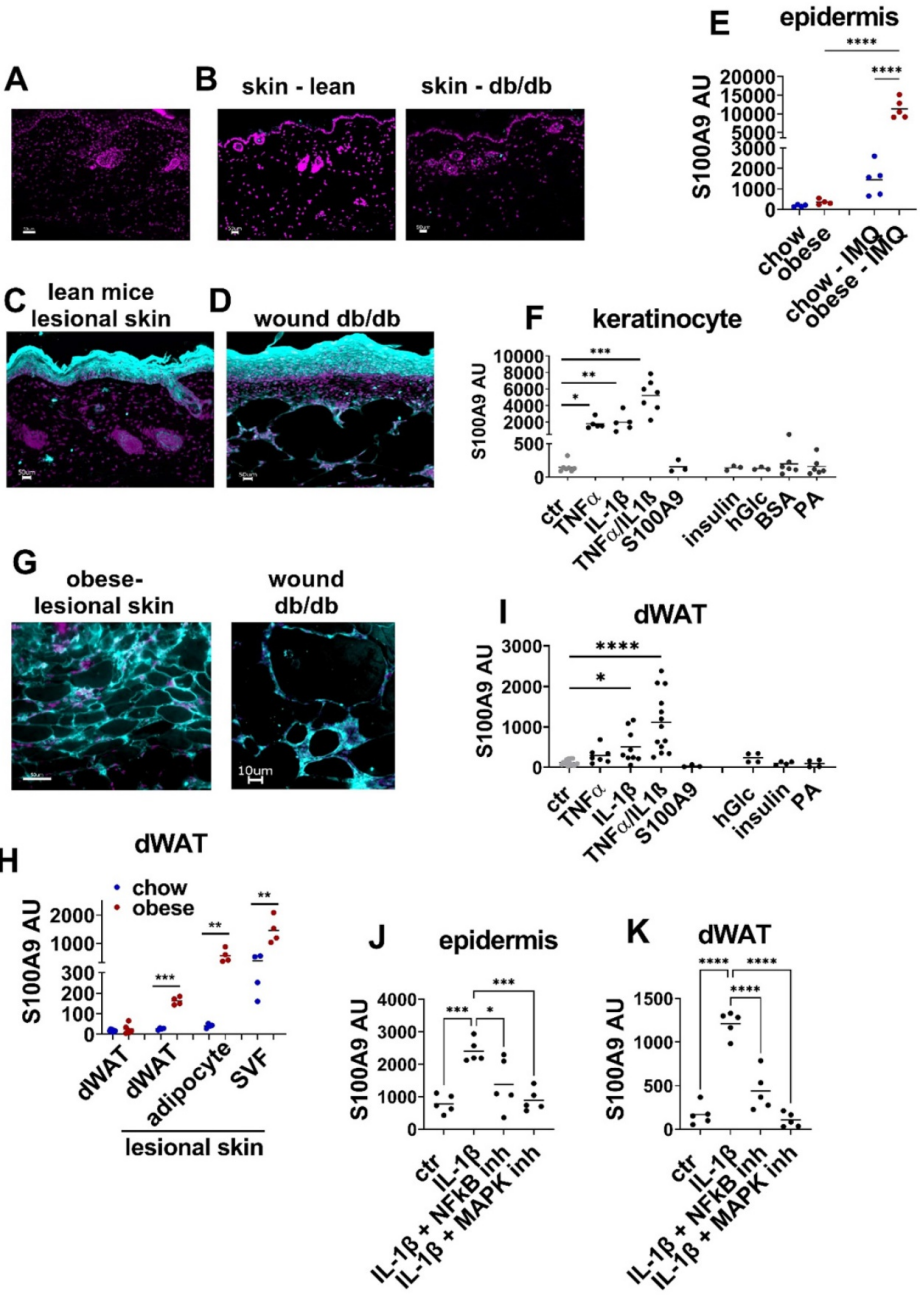
** S100A9 is expressed in keratinocytes and dermal white adipose tissue during inflammation and tissue repair. A-D)** Detection of S100A9 (turquoise) by immunofluorescence staining. Nuclei were stained with DAPI (violet). Bar 50 µm. **A)** Staining with isotype control antibody. **B)** S100A9 in healthy skin of lean wildtype mice and of *db/db* mice. **C)** S100A9 in imiquimod-induced inflamed skin of wildtype mice at d4. **D)** S100A9 in wounds of *db/db* mice at d9. **E)** Male C57BL/6 mice were fed with high fat diet or chow diet for 15 (obese) weeks. Skin inflammation was induced by one topical application of imiquimod (IMQ). Relative gene expression of S100A9 in epidermal cells of chow and obese mice and of lesional skin of chow (chow-IMQ, blue) and obese mice (obese-IMQ, red) at day 4 of inflammation.** F)** Detection of S100A9 relative gene expression by quantitative PCR in cultured primary murine keratinocytes stimulated with 10 ng/ml TNFα, 10 ng/ml IL-1β, 10 ng/ml TNFα/IL-1β, 1.5 µg/ml S100A9, 1 µg/ml insulin, 25 mM glucose or 500 µM palmitic acid (PA) complexed with BSA or BSA alone**. G)** Detection of S100A9 in dWAT areas in inflamed skin of obese mice and in wounds of *db/db* mice by immunofluorescence staining (turquoise). Nuclei were stained with DAPI (violet). **H)** Relative S100A9 gene expression in dWAT from chow (blue) and obese (red) and in dWAT, adipocytes and stromal vascular fraction (SVF) isolated from lesional skin of imiquimod treated chow and obese mice at day 6.** I)** *Ex vivo* skin cultures with biopsies of wildtype mice were stimulated with 10 ng/ml TNFα, 10 ng/ml IL-1β, 10 ng/ml TNFα/IL-1β, 1.5 µg/ml S100A9, 1 µg/ml insulin, 25 mM glucose or 500 µM palmitic acid (PA) complexed with BSA for 24h. Relative gene expression of S100A9 was detected in dWAT isolated from the skin. **J/K)** Skin biopsies from lean chow-fed mice were stimulated with IL-1β in the presence of NFkB inhibitor (BMS-345541, 4 µM) or MAPK inhibitor (Cobimitinib, 0.2 µM). Relative S100A9 gene expression in the epidermis (J) and dWAT (K) was detected by quantitative PCR. Each dot represents one mouse. Mean is indicated. ANOVA with multiple comparisons or unpaired t-test: *P < 0.05, **P < 0.01, ***P < 0.001.

To elucidate the cellular sources and underlying mechanisms of amplified S100A9 expression in inflammation and repair processes in conditions of obesity we analyzed S100A9 expression in our skin inflammation and skin wound healing models. Immunofluorescence analysis of skin sections reveals barely detectable S100A9 expression in healthy skin of lean wildtype mice and of *db/db* mice (Figure 5B). Upon induction of inflammation by IMQ or upon wounding S100A9 is highly induced in the epidermal compartment (Figure 5C-D). Gene expression analysis of the epidermal compartment confirms low S100A9 expression in the epidermis of healthy skin of chow and obese mice and an induction of S100A9 expression in the epidermis of IMQ-treated lesional skin, which is significantly higher in obese mice in comparison to lean control mice (Figure 5E). To understand the epidermal overexpression of S100A9 in obesity, we stimulated keratinocytes with obesity-associated mediators including metabolic factors such as SFA (BSA-complexed palmitic acid (PA)), insulin, high glucose (25 mM), with inflammatory factors (TNFα, IL-1β) that are increased due to the low grade inflammatory state in obesity, or with S100A9 to control for auto/paracrine effects. Consistent to the immunofluorescence staining and epidermal gene expression analysis of healthy skin unstimulated keratinocytes express low levels of S100A9 (Figure 5F). Neither S100A9, nor high glucose concentrations, insulin and PA stimulated S100A9 expression (Figure 5F). However, TNFα and IL-1β each stimulate S100A9 expression in keratinocytes while combined stimulation of TNFα and IL-1β induce a synergistic effect (Figure 5F).

Tissue staining of wounds of *db/db* mice and of IMQ-treated skin of obese mice suggest that the dermal white adipose tissue (dWAT) might be an additional yet unrecognized source of S100A9 during inflammation or tissue repair (Figure 5G). To confirm the immunofluorescence staining data we isolated dWAT from untreated and IMQ-treated skin of lean and obese mice. S100A9 is expressed at very low levels in the dWAT of untreated skin of both lean and obese mice (Figure 5H). Consistent to the immunofluorescence staining, we observed a stimulation of S100A9 gene expression in dWAT of obese mice upon induction of inflammation by IMQ (Figure 5H). In contrast, S100A9 gene expression in dWAT from lesional skin of lean mice remains low (Figure 5H). Separate analysis of adipocytes and the stromal vascular fraction (SVF) of lesional skin indicates that S100A9 is expressed in both compartments of the dWAT in obese mice (Figure 5H). It further shows that only adipocytes from obese mice express high levels of S100A9 upon induction of inflammation while S100A9 expression in adipocytes of lean mice remains low even in inflammatory conditions.

To extend our analysis regarding the expression pattern of S100A9 in inflamed skin we investigated its expression in other skin cells by flow cytometry analysis of cells after digestion of lesional skin. Analyses of viable, CD45-negative cells show that endothelial cells (detected by CD31) and fibroblasts (detected by CD90) express only very low level of S100A9 protein. Gating on CD45+/Ly6G-/F4/80+ cells indicate that macrophages also express S100A9 protein only on a low level (Figure S6).

To get insights into the regulation of S100A9 expression in the dWAT, skin of lean wildtype mice was stimulated *ex vivo* with obesity-associated mediators as described for keratinocytes and S100A9 gene expression in the dWAT was analyzed (Figure 5I). Dermal WAT in unstimulated skin expresses low levels of S100A9, which are not further increased in skin cultures with high glucose concentration, with insulin or PA but also not with S100A9. Stimulation with IL-1β or TNFα increased S100A9 expression in the dWAT. As observed in keratinocytes, co-stimulation with IL-1β and TNFα induce a synergistic upregulation of S100A9 in the dWAT fraction of the cultured skin (Figure 5I).

To understand the IL-1β-induced S100A9 expression in epidermis and dWAT *ex vivo* skin explants were stimulated with IL-1β in the presence of inhibitors of the MAPK pathway and NFkB pathway, two main signaling cascades downstream of IL-1 receptor. Blocking the NFkB pathway by BMS-345541 partially reverse IL-1β-induced S100A9 expression in the epidermis and dWAT while blocking the MAPK pathway by Cobimitinib almost completely abrogate the IL-1β-induced S100A9 expression in the epidermis and dWAT (Figure 5J-K).

Taken together, S100A9 is induced in the epidermis and dWAT during inflammation. In conditions of obesity this induction of S100A9 is highly increased in both skin departments. IL-1β and TNFα were identified as inducers of S100A9 in epidermis and dWAT while obesity-associated factors such as high glucose, insulin or SFA do not affect S100A9 expression. We therefore supposed an indirect mechanism of S100A9 overexpression during inflammation in obesity that is mediated by macrophages as one important source of TNFα and IL-1β.

**Figure 6:**
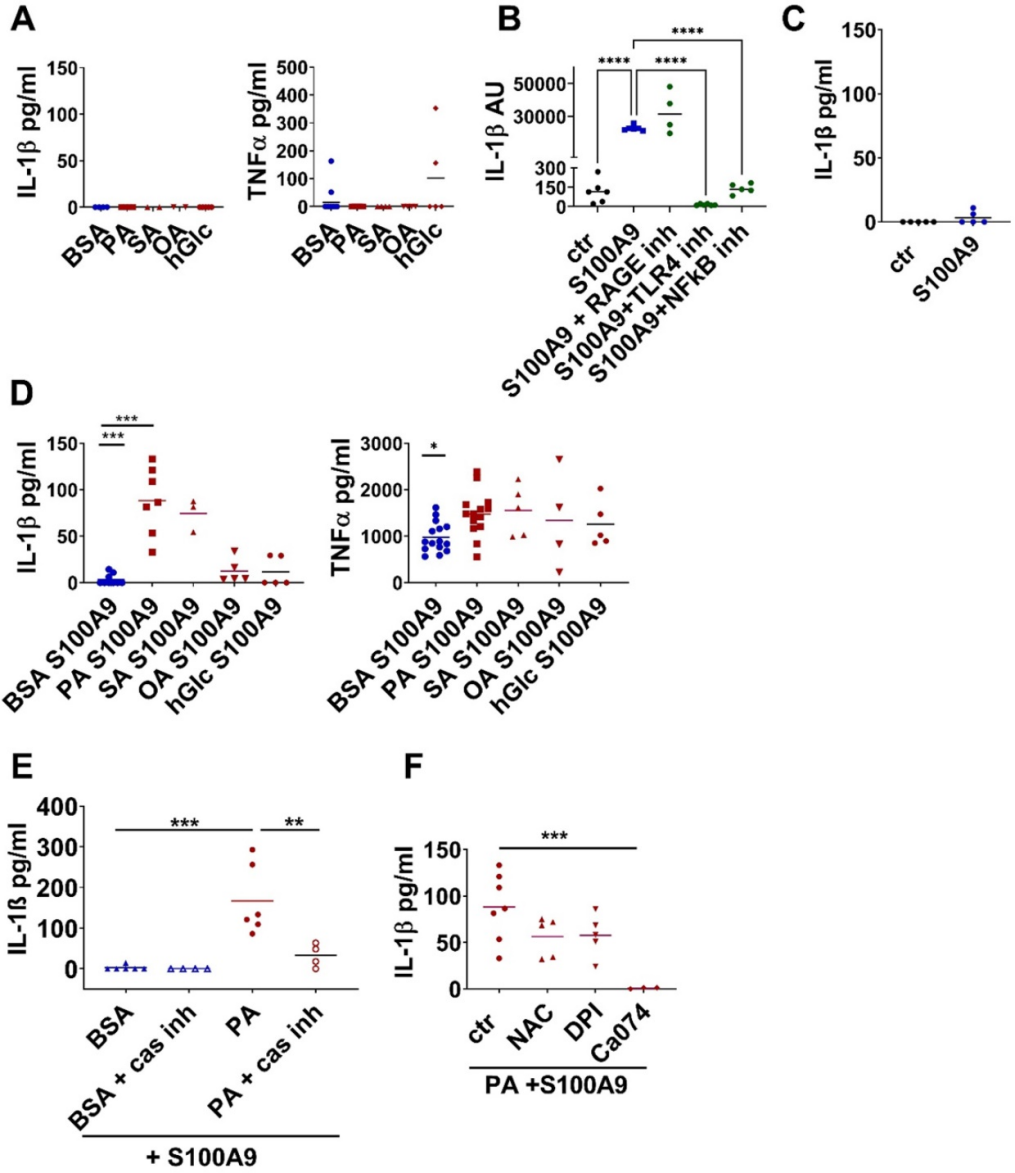
** S100A9 induces IL-1**β** secretion in macrophages in the presence of saturated fatty acids. A)** Myeloid peritoneal cells from lean chow-fed mice were incubated with 500 µM BSA-complexed PA, stearic acid (SA), or oleic acid (OA), BSA alone or 25 mM glucose (hGlc). IL-1β and TNFα levels within the supernatants were detected by ELISA. **B/C)** Myeloid peritoneal cells from lean chow-fed mice were incubated with S100A9 in the presence or absence of TLR4 signaling inhibitor (CLI-095, 1 µM), RAGE antagonistic peptide (ELKVLMEKEL, 10 µM), NFkB inhibitor (BMS-345541, 4 µM). **B)** Relative IL-1β gene expression detected by quantitative PCR; **C)** IL-1β levels in the supernatant detected by ELISA. **D)** Myeloid peritoneal cells from lean chow-fed mice were incubated with 500 µM BSA-complexed PA, stearic acid (SA), or oleic acid (OA), or 25 mM glucose followed by stimulation with S100A9. IL-1β and TNFα levels within the supernatants were detected by ELISA. **E/F)** Myeloid peritoneal cells from lean chow-fed mice were incubated with 500 µM PA-BSA complexes or BSA alone followed by stimulation with S100A9. Inflammasome activation was inhibited by addition of **E)** 25 µg/ml casapase-1 inhibitor (Ac-YVAD-cmk) or **F)** 20 mM N-acetyl-L-cysteine (NAC), 10 µM diphenyliodoniumchloride (DPI) or 5 µM Ca074 Me. IL-1β was detected after 24 h by ELISA. Each dot represents one mouse. Mean is indicated. ANOVA with multiple comparisons: *P < 0.05, **P < 0.01, ***P < 0.001.

First, we checked whether metabolic factors such as high glucose or SFA as found in diabetic obese patients directly enhance TNFα and IL-1β secretion from macrophages. As shown in Figure 6A SFA and high glucose do not stimulate TNFα and IL-1β in peritoneal cells isolated from healthy, lean control mice. Next, we stimulated peritoneal cells with S100A9 as one of the most prominent early inflammatory factors to mimic their activation in an early inflammatory response. S100A9 induces IL-1β mRNA expression in a TLR4-NFkB dependent manner. As shown in Figure 6B S100A9-induced IL-1β mRNA expression is completely prevented upon blocking of TLR4 by CLI-095 or NFkB by BMS-345541. Blocking RAGE by RAGE antagonistic peptide does not prevent IL-1β expression. Importantly, S100A9 alone is not able to induce IL-1β secretion (Figure 6C). To mimic an early inflammatory response in an obese setting peritoneal cell from healthy, lean control mice were co-stimulated with S100A9 and high concentration of FFA and glucose since both are elevated in obesity. Co-stimulation of S100A9 with high glucose had no effect on the release of both cytokines (Figure 6D). In contrast, in the presence of SFA S100A9 induces IL-1β release from macrophages (Figure 6D). In detail, peritoneal cells were co-stimulated with S100A9 and one of the following BSA-complexed FFA: the SFA PA, the SFA stearic acid (SA), or the monounsaturated fatty acid (MUFA) oleic acid (OA), while co-stimulation with BSA served as control. Stimulation with S100A9 in the control setting resulted in the secretion of TNFα while no secretion of IL-1β was induced (Figure 6C-D). Interestingly, stimulation with S100A9 in combination with SFA slightly increased S100A9-mediated TNFα release (Figure 6D) but, of note, it resulted in the induction of IL-1β release (Figure 6D). Precisely, S100A9 in the presence of the SFA PA or SA but not in the presence of the MUFA OA induced secretion of IL-1β from the peritoneal cells (Figure 6D). Combined stimulation with S100A9 and SFA amplified IL-1β secretion also in human blood-derived monocytes (Figure S7). Those were cultured for 24 h with M-CSF and stimulated with S100A9 in the presence of BSA-complexed PA, SA or OA as described for the mouse cells. Secretion of IL-1 β was induced after stimulation with S100A9 only in the presence of SFA but not with S100A9 alone or in combination with OA (Figure S7A). Consistently, human monocytes incubated with serum containing high levels of total FFA secreted significantly more IL-1β upon S100A9 stimulation than when incubated with low FFA serum (Figure S7B-C).

In order to investigate whether the SFA/S100A9-mediated IL-1β release involves an inflammasome-dependent pathway we stimulated mouse peritoneal cells with PA and S100A9 and added Ac-YVAD-cmk, an inhibitor of caspase-1, the enzyme that cleaves pro-IL-1β into its active form. Indeed, blocking the inflammasome activation by Ac-YVAD-cmk abolished the secretion of IL-1β by S100A9 in combination with PA (Figure 6E). Further analysis revealed that lysosome damage contributes to inflammasome activation since inhibition of cathepsin B by Ca074 Me also abolished PA-S100A9-mediated IL-1β release (Figure 6F). Blocking reactive oxygen species (ROS) by either N-acetyl-L-cysteine (NAC) or diphenyliodonium chloride (DPI) only slightly reduced IL-1β secretion (Figure 6F) suggesting that ROS activation plays only a minor role. Taken together, S100A9 induces IL-1β gene expression in macrophages via TLR4 activation and NFkB downstream signaling. In the presence of SFA, S100A9 signaling contributes to the activation of caspase-1-dependent inflammasome and the secretion of IL-1ß from macrophages.

**Figure 7:**
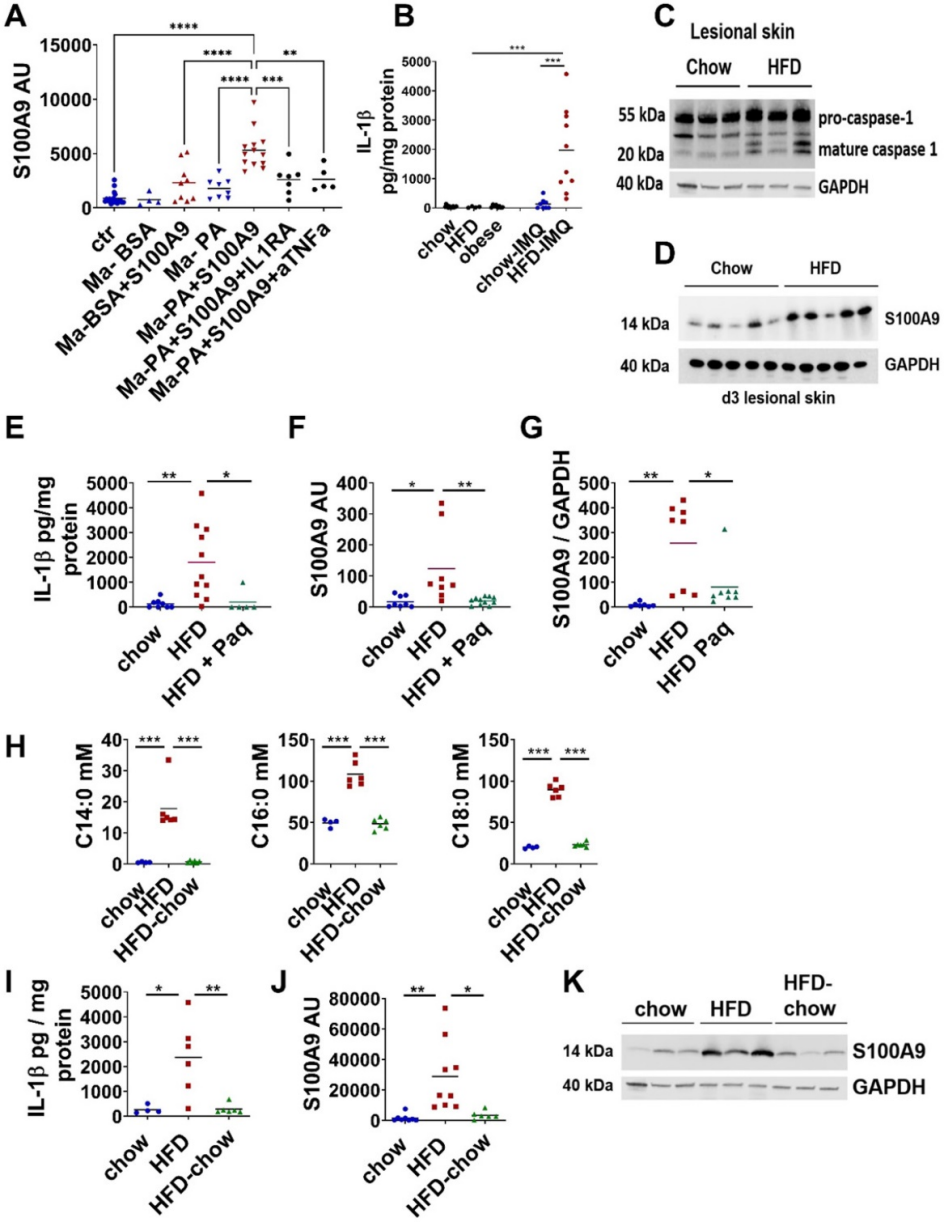
** Saturated fatty acids in combination with S100A9 drive S100A9 overexpression in skin inflammation via induction of IL-1β secretion in macrophages. A)** Skin biopsies from lean chow-fed mice were cultured with supernatants of PA-, BSA-, PA/S100A9- and BSA/S100A9-stimulated myeloid cells (Ma) or medium. IL-1β and TNFα were neutralized by pre-incubation with 200 ng/ml IL-1 receptor antagonist (IL1RA) and 1 µg/ml anti-TNFα antibody, respectively. Relative expression of S100A9 in the skin was detected by quantitative PCR. **B-D)** Male C57BL/6 mice were fed with high fat diet (HFD, red) or chow diet (blue) for 6 weeks. Skin inflammation was induced by one topical application of imiquimod on the shaved back. **B)** Detection of IL-1β in untreated skin of lean, HFD and obese mice and in lesional skin (day 4) of chow and HFD by ELISA. IL-1β concentration related to protein content is shown. **C)** Detection of mature caspase in lesional skin (day 4) by Western Blot. GAPDH was used as loading control. **D)** Detection of S100A9 in lesional skin at day 4 by Western Blot. GAPDH was used as loading control.** E-G)** Male C57BL/6 mice were fed with chow diet (blue) or high fat diet (HFD, red) for 6 weeks. Skin inflammation was induced as in B-D. Paquinimod was applied 10h after imiquimod (HFD+Paq; green). Mice were analyzed at day 4. **E)** Detection of IL-1β in lesional skin by ELISA. IL-1β concentration related to protein content is shown.** F)** Relative S100A9 gene expression in the epidermis detected by quantitative PCR. **G)** Detection and densitometric evaluation of S100A9 by Western Blot. GAPDH was used as loading control. **H-K)** Male C57BL/6 mice received either HFD for 6 weeks (red), HFD for 5 weeks followed by chow diet for one week (green), or chow diet for 6 weeks (blue). Skin inflammation was induced as in B-D. **H)** Concentrations of saturated fatty acids in mM, C14:0 - myristic acid, C16:0 - palmitic acid, C18:0 - stearic acid. **I)** Detection of IL-1β in lesional skin by ELISA. IL-1β concentration related to protein content. **J)** Relative gene expression of S100A9 in the epidermis detected by quantitative PCR. **K)** Detection of S100A9 in lesional skin by Western Blot. GAPDH was used as loading control. Each dot represents one mouse. Mean is indicated. ANOVA with multiple comparisons: *P < 0.05, **P < 0.01, ***P < 0.001.

Next, we investigated the consequences of SFA/S100A9-mediated IL-1β secretion from macrophages on S100A9 expression in the skin. We stimulated peritoneal cells with S100A9 and PA or BSA, respectively, to generate conditioned supernatants. We then used these supernatants to perform *ex vivo* cultures with skin biopsies from healthy mice and analyzed the expression of S100A9 in the skin. As shown in Figure 5F-I S100A9 did not modulate its own expression in keratinocytes or dWAT excluding an effect of S100A9 in the macrophage supernatant on S100A9 expression. Supernatants of BSA- or PA-stimulated macrophages containing no TNFα and IL-1β did not significantly affect S100A9 expression in the skin (Figure 7A). Supernatants of BSA/S100A9-stimulated cells containing only TNFα slightly stimulated S100A9 expression (Figure 7A). Importantly, only supernatants of PA/S100A9-activated macrophages containing both IL-1β and TNFα (Figure 6D) significantly increased S100A9 expression in the skin (Figure 7A). Blocking of IL-1β or of TNFα by addition of IL-1RA or of TNFα neutralizing antibody to the supernatants of PA/S100A9-activated macrophages inhibited the induction of S100A9 expression in the skin (Figure 7A) stressing out the importance of IL-1β and TNFα in this process To analyze the contribution of keratinocytes and dWAT to the increase of S100A9 expression upon stimulation with supernatants of PA/S100A9-activated macrophages the epidermal compartment and dWAT were separated after *ex vivo* skin culture. As shown in Figure S8 PA/S100A9-activated macrophages stimulated S100A9 expression in the epidermis and dWAT in an IL-1β-dependent manner as observed in total skin. In summary, these data show that SFA in combination with S100A9 induce IL-1β which in turn amplifies S100A9 expression in the skin.

To prove the mechanistic link between SFA, S100A9 overexpression and inflammasome activation in an inflammation context *in vivo,* we analyzed IL-1β expression and release in our short-HFD skin inflammation model in which HFD-fed mice present increased levels of serum SFA but unaltered carbohydrate metabolism (fasting glucose and glucose tolerance similar to control mice (Figure S1)). Indeed, we detected increased IL-1β protein in the inflamed skin of short-HFD mice in comparison to chow control mice (Figure 7B). We also analyzed IL-1β in healthy skin of lean, HFD and obese mice and found almost no IL-1β protein. This further supports our *in vitro* finding, that altered metabolic factors alone do not induce an increase in IL-1β. Consistent to the *in vitro* data showing inflammasome activation by S100A9 in the presence of SFA we found increased levels of mature caspase-1 in the presence of elevated SFA in the inflamed skin of these mice (Figure 7C). In parallel to the increase of IL-1β protein, expression of S100A9 protein is increased in inflamed skin (Figure 7D) in the short-HFD-mice.

Finally, to prove the importance of S100A9 and elevated SFA as driver of the IL-1β-mediated upregulation of S100A9 we blocked their activity during IMQ-driven skin inflammation in short-HFD. First, we analyzed short-HFD mice after blocking S100A9 function by paquinimod at day 4 when inflammation had established and inflamed skin of short-HFD mice is characterized by elevated levels of IL-1β and S100A9. Indeed, release of IL-1β and gene and protein expression of S100A9 in inflamed skin of short-HFD-mice treated with paquinimod is reduced to the level of chow control mice (Figure 7E-G). This demonstrates that blocking of S100A9 activity prevents sustained expression of IL-1β and S100A9 in an ongoing inflammatory response under HFD conditions. Next, we reduced the SFA level in the mice by a dietary intervention approach. We fed two groups of mice with HFD for 5 weeks. Then one group switched to chow control diet for one week while the second group continued on receive HFD. A third control group received chow diet for 6 weeks. Diet change for one week reduce serum SFA to the level of chow mice (Figure 7H). We then induced a skin inflammation by applying IMQ in all three groups of mice. Again, we analyzed inflamed skin at day 4. In mice from the dietary intervention group lacking elevated SFA we observed a reduction of IL-1β protein secretion and S100A9 gene and protein expression to the levels of the chow control group (Figure 7I-K). This demonstrates the functional role of SFA to the overexpression of S100A9 in skin inflammation in obesity.

Since our data indicate that SFA are one important driver of S100A9 overexpression in obesity we investigated whether reduction of SFA restores M2 differentiation. Consistent with the lack of S100A9 overexpression (Figure 7J-K), short-HFD mice with reduced SFA show restored M2-like macrophage differentiation in the inflamed skin and reduced skin inflammation (Figure S9A-D). Similar results were observed in obese mice. Diet change for one week has no significant effect on the weight of the mice (Figure S9E) but significantly reduce levels of serum SFA (Figure S9F) although not to the level of chow control mice as observed in the short-HFD model. However, dietary reduction of SFA in the obese mice reduces epidermal expression of S100A9 in the inflamed skin (Figure S9G). At the same time, obese mice show improved M2-like macrophage differentiation, less severe skin inflammation and less ear swelling after diet change, but not to the level of control mice (Figure S9H-J).

**Figure 8:**
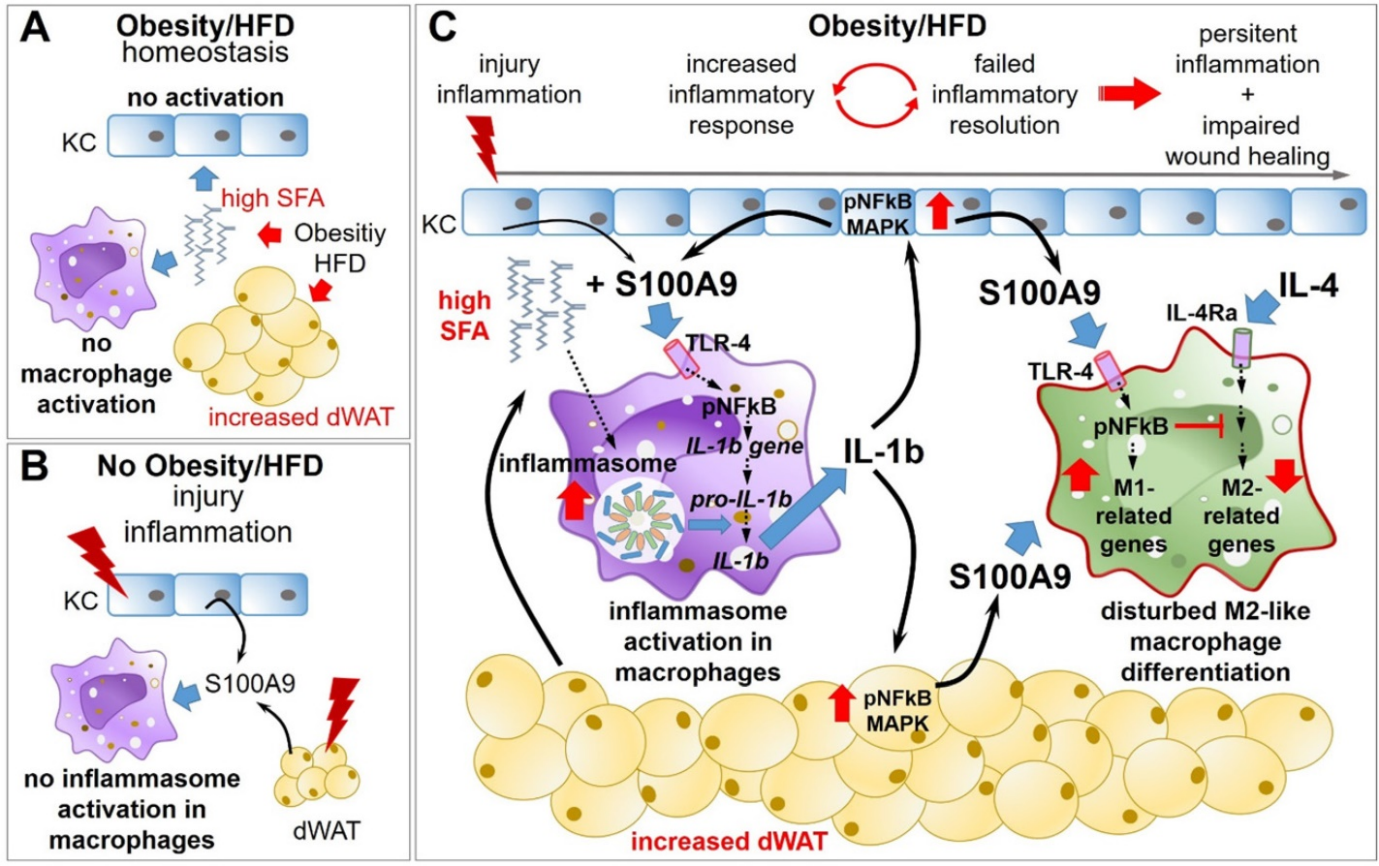
** Overexpression of S100A9 in obesity impairs M2-like macrophage differentiation resulting in amplified and sustained inflammation as well as impaired tissue repair. A)** Obesity and high fat diet result in elevation of saturated fatty acids (SFA), which have no impact on keratinocytes (KC) in homeostatic conditions. **B)** S100A9 release upon injury/inflammation in the absence of elevated SFA does not promote inflammasome activation in resting macrophages/monocytes. **C)** Upon injury/inflammation S100A9 is released by keratinocytes and dermal white adipose tissue (dWAT). S100A9 binding to TLR4 and activation of NFkB induces gene expression of IL-1β in macrophages. In the presence of high SFA, elevated by obesity or HFD, inflammasome is activated in the cells resulting in IL-1β release. Macrophage-derived IL-1β in turn amplifies S100A9 expression in epidermis and dWAT via NFkB and MAPK signaling pathways, initiating a perpetuating S100A9 response in inflammation in obese skin. S100A9 impairs an appropriate macrophage activation and polarization via TLR4-NFkB signaling preventing resolution of skin inflammation that finally contributes to a sustained and amplified skin inflammation and impaired tissue repair.

All together our *in vitro* and *in vivo* data demonstrate that sustained overexpression of S100A9 in skin inflammation in obesity is mediated by SFA that initiate and drive a positive feedback loop between skin cells and the dermal macrophage compartment via the induction of an inflammasome-dependent IL-1β release (Figure 8).

## Discussion

Well-balanced differentiation and activation of macrophage subsets plays an important role for the maintenance of tissue homeostasis, adequate immune responses, and tissue repair. The high plasticity of macrophages allows them to adapt their phenotype and response to environmental signals. Thus, alteration of the environment such as in obesity has detrimental effects on the fine-tuned processes of macrophage differentiation resulting in an imbalance of M1/M2 macrophages in adipose tissue (AT) 10. In addition to the effect of AT accumulation on glucose tolerance and insulin sensitivity, obesity amplifies a wide range of inflammatory diseases in multiple organs and attenuates wound repair 22-24,29,41-43. However, mechanistic studies are required to improve the understanding of the relationship between obesity, inflammation and tissue repair.

In the present study, we show that obesity-associated overexpression of S100A9 in addition to its known chemotactic and pro-inflammatory actions impairs macrophage differentiation that contributes to amplification of skin inflammation and delayed resolution as well as impaired skin repair in obesity.

S100A9 belongs to the family of danger-associated molecular patterns induced upon infection, injury or inflammation to initiate the first rapid inflammatory response 34,36. Neutrophils are an important source of S100A9 especially in the initial phase of inflammation when infiltration of neutrophils is dominant. Since release of S100A9 represents an important trigger for the onset of inflammation and immune processes its expression requires tight control. Loss of regulatory mechanisms has been shown to result in fatal TNFα-driven inflammation 33. Known immunoregulatory functions of S100A9 contributing to inflammation in this respect include stimulation of leukocyte recruitment and activation of pro-inflammatory cytokine expression in immune and tissue-resident cells 33. Interestingly, in all our models, we observed an overexpression of S100A9 in obese mice, short-HFD mice or *db/db* mice at times when M2 activation and inflammatory resolution had already occurred in the respective control mice suggesting an unknown role of S100A9 in the control of macrophage activation. *In vitro* experiments with murine peritoneal macrophages demonstrate that addition of exogenous recombinant S100A9 disturbed IL-4-mediated differentiation into M2-like macrophages. These cells showed a gene expression profile with a mixture of typical M1 and M2 genes but important genes of M2-macrophage differentiation including relmα and CD301 44 were almost completely blocked.

It has been described that S100A8 and S100A9 exist as homodimers but preferentially form the S100A8/A9 heterodimer (also called calprotectin) in the presence of Zn2+ and Ca2+ 18. However, there are conflicting results on the functional impact of the homodimers S100A8 and S100A9 and the heterodimer. In the present study we show that S100A8 homodimer or S100A8/A9 heterodimer are not sufficient to alter M2-like macrophage differentiation while S100A9 homodimer efficiently inhibits M2-like macrophage differentiation in a TLR4-NFkB dependent way. Importantly, Källberg et al. describe the induction of S100A9 homodimer *in vivo* in situations of tumor burden or inflammatory challenge 45. They detected S100A9 monomers, S100A8/S100A9 heterodimers and S100A9 homodimers intracellularly in spleen cells of tumor bearing animals while only S100A9 monomers and homodimers were secreted. Using mass spectrometry, non-covalent and covalent homodimers of mouse S100A9 have been identified 46. Consistent to our data, Björk et al. demonstrated that S100A9 binds to immobilized TLR4 while S100A8 shows only weak interaction 40. In addition, it was shown that recombinant S100A9 homodimers induce the expression of pro-inflammatory and osteogenic factors in human macrophages 47. In contrast, Vogl et al. describe that S100A8 is able to induce TNFα expression in phagocytes via TLR4 recognition whereas S100A9 had no effect. Based on these findings they suggest that S100A8 is the active component in the S100A8/A9 heterodimer, and that S100A9 modulates the activity of its binding partner 48. A further study shows that both S100A8 and S100A9 induce cytokine and chemokine release in human tendocytes, and that S100A9 was more effective than S100A8 49. These different data might be due to cell-specific and species-specific effects of the different S100A8 and A9 forms or different environmental conditions such as the presence of specific cations or pH. It becomes more and more evident that S100 proteins exist *in vivo* in multiple forms. This plasticity might contribute to their diverse and sometimes opposite functions 46.

TLR4 and RAGE have been described as receptors for S100A9 40. S100A9-mediated cytokine release in human THP-1 cells and in mouse bone marrow-derived dendritic cells involves TLR4 signaling 50. Receptor-blocking reveals that S100A9-mediated dysregulation of macrophage differentiation is dependent on S100A9 binding to TLR4 while RAGE does not contribute to this effect.

Proteome analysis indicates that S100A9 interfere with a multitude of pathways during M2-like macrophage differentiation underlining the impact of S100A9 on macrophage differentiation. Thus, S100A9 induces several pro-inflammatory pathways while Stat6, one of the master regulators of M2-like macrophage differentiation 44 was not affected. It is described that other factors also interfere with M2 differentiation independent of Stat6 signalling, such as Klf4 51. Klf4-deficient macrophages show increased expression of pro-inflammatory genes associated with enhanced M1 macrophage polarization 51 as observed by us in macrophages after treatment with S100A9. The S100A9-mediated induction of a pro-inflammatory phenotype in M2-macrophages and the inhibition of M2-like gene signatures include mainly NFκB activation as described for pro-inflammatory effects of S100A9 in other cells 34,52.

Consistent to our *in vitro* findings of impaired M2-differentiation by S100A9, we observed disturbed M2-like macrophage differentiation in skin inflammation and wound healing when S100A9 expression is pathologically increased. A crucial role for S100A9 in the control of macrophage function was also described by Dessing et al. They observed an increased activity of M2-like macrophages associated with fibrosis in a renal ischemia/reperfusion injury model in S100A9-knockout (KO) mice 53. They further show that S100A9-deficient macrophages from S100A9-KO mice are predisposed to sustain a M2-like phenotype 53. This suggests an additional intrinsic role of S100A9 in the control of macrophage functions. However, in this study we address the role of exogenous S100A9 on macrophage activation and reveal an unrecognized function of exogenous S100A9 in the control of M2 macrophage differentiation. Restoration of M2-like macrophage activation upon blocking of S100A9 in enhanced obesity-associated skin inflammation in obese mice and short-HFD mice and impaired wound healing in *db/db* mice prove the novel role of S100A9 in the control of macrophage polarization. S100A9 was blocked by paquinimod which inhibits the binding of S100A9 to its receptors, TLR4 and RAGE 40. Efficacy of S100A9 blocking by paquinimod was already shown in several mouse models of inflammatory diseases including experimental autoimmune encephalitis 54, liver fibrosis 55, lupus-prone MRL-lpr/lpr mouse model 56, and skin fibrosis 57. In line with these studies we observed less severe skin inflammation in obese mice and short-HFD mice after treatment with paquinimod. Since S100A9 is crucial for the manifestation of an inflammatory response 34 we administered paquinimod with 10 h delay to the application of IMQ in the skin inflammation models. Thus, inhibition of S100A9 activity took effect when skin inflammation had established and had no impact on already described S100A9 function in early inflammation. Since M2-differentiation is a crucial step in tissue repair we analyzed skin wound healing upon blocking S100A9 in diabetic *db/db* mice which are characterized by a disturbed M1-M2 balance and impaired wound healing, resembling some features of non-healing wounds in diabetic patients 58. As observed in our skin inflammation models, blocking S100A9 restored disturbed macrophage differentiation during wound healing in *db/db* mice. In parallel, wound repair was improved in these mice. Again, we administered paquinimod two days after wounding to avoid affecting the known chemotactic activity of S100A9. Hence, we assume that less severe skin inflammation and improved wound healing in paquinimod-treated mice are attributed to the restored activation of M2-macrophages due to blocking of S100A9 activity in the later phase of inflammation. We acknowledge that we do not provide direct evidence of disturbed M2-macrophage differentiation as ultimate cause for increased skin inflammation and delayed wound repair in obese mice. However, restored activation of M2 macrophages after blocking S100A9 indicates an association. In this context, several studies have demonstrated the decisive role of M2 macrophages in regulating inflammation and tissue repair by improving inflammatory processes and wound healing through targeted support of M2 macrophage activation 59,60. In conclusion, our data indicate that a dysregulated S100A9 overexpression as observed in obesity has detrimental effects on macrophage differentiation and thus on inflammation and tissue repair. Consistently, pathogenic up-regulation of S100A9 has been observed in various inflammatory diseases 18,34,35. Understanding the mechanisms of obesity-driven S100A9 overexpression might open new opportunities to interfere with dysregulated inflammation and tissue repair in obesity.

In the present study we found almost no expression of S100A9 in skin of lean, obese and *db/db* mice suggesting that obesity per se does not affect S100A9 expression. S100A9 secretion is induced during infection, injury and inflammation. Neutrophils and monocytes have been described as cellular source (34). However, in the present study we focus on the effect of S100A9 on M2-like macrophage differentiation which takes place at a time when neutrophils and monocytes had been mostly removed suggesting that S100A9 derived from these cells might play only a minor role in the control of M2-like macrophage differentiation. Consistent with studies showing high expression of S100A9 in keratinocytes in psoriatic skin lesions and in wounds 35,37, we found high epidermal S100A9 expression in skin inflammation and during wound healing in our mice that is further exacerbated by obesity. We further describe the dWAT as a new and additional important source of S100A9 in inflammation and tissue repair in the skin and demonstrate that S100A9 expression is strongly enhanced in the dWAT of obese mice upon induction of an inflammatory response in the skin. Both, adipocytes and SVF express S100A9 in dWAT of inflamed skin. In lean mice predominantly the SVF express S100A9 after induction of inflammation while the expression level in adipocytes is very low. In contrast to this, adipocytes from the dWAT in obese mice present a very high expression of S100A9 in the inflamed skin, while the expression of S100A9 in SVF is only slightly increased in comparison to SVF in lean mice. This suggests adipocytes as a main source of increased S100A9 in inflammation in the dWAT in obese skin. It further indicates that in conditions of obesity the activation state of adipocytes in the dWAT is changed resulting in the expression of S100A9 in inflammatory settings. Indeed, S100A9 expression has been already described in visceral adipose tissue 61. Expression of S100A8 and S100A9 was found in both adipocytes and the SVF isolated from human visceral AT 62. In addition, increased gene expression of S100A8 and S100A9 in visceral AT and increased concentrations of S100A8/A9 complexes in the circulation have been observed in obese patients and correlated with the obesity associated low-grade inflammation in the patients 62. However, in addition to energy storage the function of adipocytes differ remarkably between different depots. For instance, mammary adipose tissue expands and involutes during lactation, whereas epicardial white adipocyte tissue feeds free fatty acids to adjacent myocardial cells. Adipocytes in the dWAT display functions unique to the skin such as antimicrobial defense, hair cycle regulation, wound healing, and temperature regulation 63. Zhang et al describe that dermal adipocytes are a distinct class of white adipocytes with high plasticity 64. RNA-Seq of dermal adipocytes and adipocytes isolated from the subcutaneous layer revealed significant differences between these 2 types of adipocytes 64. The present study shows to our knowledge for the first time the expression and regulation of S100A9 in the dWAT.

Typical obesity-associated mediators such as high SFA, glucose or insulin do not directly change S100A9 expression in keratinocytes and the dWAT. However, TNFα and IL-1β stimulated S100A9 expression and the combination of both resulted in a synergistic effect. Various pathways and transcription factors including NFkB, MAPK, p53, HIF1α, Hbx, and Stat3 have been described to regulated S100A9 expression 65-69. Here, we show that IL-1β induced expression of S100A9 in the skin is mediated via NFkB and MAPK activation. The fact that obesity-associated mediators do not induce S100A9 expression but TNFα and IL-1β do so, suggests an indirect mechanism for the increased S100A9 induction in dWAT and epidermis in the inflamed skin in obesity. We show that SFA, which are increased in obesity, together with S100A9, an early danger molecule, induce IL-1β release in macrophages which in turn amplifies S100A9 expression initiating a vicious cycle of sustained S100A9 overexpression in skin inflammation in obesity. S100A9 induces IL-1β expression in macrophages via the TLR4-NFkB pathway as it was described in human THP-1 cells and in mouse bone marrow-derived dendritic cells 50. Blocking RAGE does not affect S100A9 induced IL-1β gene expression which is consistent to data from Riva et al. showing that S100A9-induced NFκB activation was completely absent in TLR4 knockout mice, whereas it was only slightly affected in RAGE knockout mice 50. In addition, S100A9 induces the activation of monocytes to release inflammatory cytokines like TNFα 33 as we also found in this study. Importantly, S100A9 stimulation alone fails to induce IL-1β secretion. However, in the presence of high SFA levels, like they are present in obesity, S100A9 induces an inflammasome-dependent secretion of IL-1β by monocytes and macrophages as shown here for both mouse peritoneal macrophages and human monocytes. Our findings are consistent with a study showing that S100A8/A9 signaling in macrophages results in IL-1β expression via the MyD88 pathway and that in the presence of secondary stimuli, such as extracellular ATP, NLRP3 inflammasome-dependent processing and secretion of IL-1β is induced 61. Blocking experiments with inhibitors of caspase-1 and cathepsin B demonstrate that the release of IL-1β involved inflammasome activation and lysosomal damage by SFA as it was described by Karasawa et al. 70. Since our *in vitro* data suggest an important role of SFA in the control of S100A9 expression we used short-HFD mice where mice were fed a HFD enriched in SFA for only 6 weeks. The short-HFD mice had a normal body weight and glucose metabolism but showed elevated serum levels of SFA. They therefore represent an excellent model to investigate the role of SFA independent of other HFD-induced metabolic changes such as hyperglycemia or insulin resistance. Indeed, we detected increased IL1-β amounts in inflamed skin of short-HFD-fed mice and increased activity of caspase-1. Various studies report the capability of SFA to activate NLRP3 inflammasome and release of IL-1β in cells in response to a stimulation with the TLR4 agonist lipopolysaccharide 31,70-72. In line with these studies, we observed an induction of IL-1β secretion in macrophages after stimulation with SFA and S100A9, which is an endogenous TLR4-agonist. Our data reveal that SFA promote inflammasome-dependent IL-1β release in skin inflammation *in vivo*. Abrogation or downregulation of IL-1β release after reduction of SFA in short-HFD-fed mice demonstrate the requirement of elevated SFA level for IL-1β promotion in this context. IL-1β is an important regulatory cytokine in the onset of inflammation processes and activates immune and tissue cells 73. Here, we show that the SFA/S100A9-mediated enhanced release of IL-1β and TNFα by macrophages promotes the expression of S100A9 in skin cells in *ex vivo* skin cultures. Consistently, sustained overexpression of S100A9 in skin inflammation in short HFD-mice and obese mice coincides with an increase of IL-1β in myeloid cells isolated from the lesional skin. Consequently, inhibition of S100A9 or reduction of SFA as one driver of S100A9 overexpression in obesity breaks this viscous cycle of S100A9 overexpression.

In summary, in obesity overexpression of S100A9 impairs appropriate macrophage activation and polarization preventing resolution of skin inflammation that finally contributes to a sustained and amplified skin inflammation and impaired tissue repair in obesity (Figure 8).

## Methods

### Mouse studies

Four weeks old male C57BL/6 mice were fed with HFD (EF R/M D12331 diet modified by Surwit; 35.7% crude fat)) or chow diet (4.5% crude fat) for 6 or 15 weeks 31. Glucose tolerance was determined after 16 h starvation. Glucose was applied intraperitoneally at 2 g/kg body weight. Blood glucose concentration was detected after 0, 15, 30, 60, and 120 min by blood glucose meter.

Skin inflammation was induced by topical application of 125 mg IMQ (Aldara, Meda GmbH, Wiesbaden, Germany) on the shaved back of C57BL/6 mice including the dorsal side of the ears. Severity score representing the sum of erythema and scaling of the back skin (0: none; 1: mild; 2: moderate; 3: severe) was determined by two independent researchers. Ear thickening was determined using a caliper. Treatment with the Q compound paquinimod (a gift from Active Biotech, AB, Lund, Sweden) was started 10 h after IMQ application. It was administered via drinking water to achieve a concentration of 20 mg/kg/d.

Wound healing study: Wounding was performed with full-thickness 6 mm punch biopsies on the back of C57BL/6 mice or *db/db* mice (13 weeks old) under anesthesia. Treatment with paquinimod (a gift from Active Biotech, AB, Lund, Sweden) at a concentration of 20 mg/kg/d was started two days after wounding. Wounds were harvested at indicated time points post wounding.

### Isolation of skin cells

Myeloid cells were isolated after enzymatic digestion of skin by 0.15 mg/ml liberase (Roche, Mannheim, Germany) and 0.12 mg/ml DNase (Sigma-Aldrich Chemie GmbH, Taufkirchen, Germany) as described 31. After 1 h myeloid cells were isolated using CD11b+ Cell Isolation Kit (Miltenyi, BergischGladbach, Germany) according to manufacturer´s instructions.

Epidermal cells were isolated upon segregation of the epidermis from dermis by overnight incubation with 0.25% trypsin (Biochrom AG, Berlin, Germany).

Dermal WAT was mechanically removed from the dermis. For isolation of SVF and dWAT dWAT was digested by 0.15 mg/ml liberase (Roche) for 1 h at 37°C. Tissue homogenate was filtered through a 70 µm filter and centrifuged at 4000 rpm for 10 min. The floating cell layer containing adipocytes was isolated and washed once. The cell pellet represents the SVF and was washed once with PBS.

### RNA preparation and quantitative real-time PCR

Total RNA from homogenized skin biopsies and cells was isolated using the Relia Prep RNA Tissue Miniprep System (Promega, Walldorf, Germany) according to the manufacturer′s protocol. cDNA was generated from a total of 500 ng RNA with LunaScript RT Supermix (NEB, Frankfurt a.M., Germany) as described in the manufacturer′s protocol. Quantitative real-time PCR was performed with LunaUniversal qPCR Mastermix (NEB) according to the manufacturer′s instructions (see Table S1 for primer sequences). All PCR products are intron-spanning. Quantitative gene expression was calculated from standard curve of cloned cDNA and normalized to the reference gene RS36 (Table S1).

### Genome-wide expression analysis

Before microarray analysis RNA integrity and concentration were examined on an Agilent Fragment Analyzer (Agilent Technologies, Palo Alto, CA, USA) using the HS RNA Kit (Agilent) according to the manufacturer's instructions. Microarray analysis was conducted at the Core Unit DNA Technologies (Faculty of Medicine; Leipzig University). cRNA was prepared from 100 ng of total RNA hybridized to Gene Chip Clariom S arrays (Thermo Fisher Scientific, Karlsruhe, Germany) according to the manufacturer's instructions. The arrays were scanned with a third generation AffymetrixGeneChipScanner 3000. For data analysis, Affymetrix Gene Chip data were extracted from fluorescence intensities and were scaled in order to normalize data for inter-array comparison using Transcriptome Analysis Console (TAC) 4.0.2 software according to manufacturer's instruction (Thermo Fisher Scientific).

### Proteomics

Mouse peritoneal cells (1×10^6^) from two different mice were isolated and cultured at 8 x 10^5^ cells/ml in RPMI 1640 medium (Thermo Fisher Scientific) containing 10% FCS (ThermoFisher Scientific), 1% penicillin/streptomycin (Biochrom AG), and 10 ng/ml M-CSF (Miltenyi) for 24 h. M2-like macrophage differentiation was induced by addition of 20 ng/ml IL-4 (Miltenyi) in presence or absence of 1.5 µg/ml recombinant murine S100A9 homodimer (R&D, Minneapolis, MN, USA). Cells were lysed with scioExtract buffer after 30 min according to the manufacture's protocol (Sciomics GmbH, Neckargemünd, Germany) and stored in liquid nitrogen. Protein labelling and analysis was performed by Sciomics GmbH. Upon labelling with scioDye 2 samples were analyzed on a scioDiscover antibody microarrays (Sciomics) targeting 1,352 different proteins with 1,821 antibodies. Afterwards microarrays were incubated with a pan-phospho-antibody. Slide scanning was conducted using a Powerscanner (Tecan, Austria). Spot segmentation was performed with GenePix Pro 6.0 (Molecular Devices, Union City, CA, USA). Acquired raw data were analyzed using the linear models for microarray data (LIMMA) package of R-Bioconductor after uploading the median signal intensities. For normalization, a Cyclic Loess normalization was applied. Differences in protein abundance or phosphorylation level between sample groups are presented as log-fold changes (logFC) calculated for the basis 2. Pathway analysis was performed with KEGG database.

### Western Blot

Skin was homogenized in ProcartaPlex® Cell Lysis Buffer (Thermo Fisher Scientific) containing 1 mM PMSF (Miltenyi) using 5 mm Stainless Steel Beads (Qiagen, Hilden, Germany) and TissueLyser LT (Qiagen). Lysates were centrifuged for 10 min at 16,000 x g, 4°C. Proteins were separated by 8-16% SDS PAGE (Biorad; Munich, Germany) and blotted onto nitrocellulose membrane. Anti-S100A9 antibody (R&D, Wiesbaden, Germany), anti-rpl26 (Sigma), anti-GAPDH (Millipore, Darmstadt, Germany), anti-p-Stat6, anti-p-Erk, anti-p-NFkB, anti-p-p38 (Cell Signaling, Frankfurt a.M, Germany) were used. After overnight incubation and washing steps anti-rabbit IRDye680 labelled antibody (Invitrogen) was added followed by washing and detection at LI-COR Odyssey Fc (LI-COR, Inc., Lincoln, NE, USA). Signals of investigated proteins were normalized to reference proteins Rpl26 or GAPDH.

### ELISA

Murine IL-1ß, TNFα, IL-6 were detected by ELISA according to the manufacturer´s protocol (Thermo Fisher Scientific) and detected using Synergy HT (BioTek, Bad Friedrichshall, Germany).

### Flow cytometry

Cells were incubated with antibody mixture including anti-F4/80-PE (BioRad), anti-Ly6G-FITC, anti-CD11b-PECy7, anti-CD45-PerCPCy5.5 and anti-Ly6C-APC or, anti-CD301-APC or anti-CD206 APC (Biolegend, San Diego, CA, USA) for 15 min and subsequently, 0.4 µl zombie dye (Biolegend) was added for 5 min. For detection of S100A9 protein intracellular staining was performed using the True-Nuclear™ Transcription Factor Buffer Set. Cells were first stained with antibody mixture including anti-CD45-PerCPCy5.5, anti-CD11b-PECy7, anti-Ly6G-APC, anti-F4/80-PE or anti-CD45-PerCPCy5.5, CD31-PE, CD90-PE-Cy7 for 15 min, washed and fixed for 45 min before intracellular staining with goat anti-S100A9 antibody (R&D) for 30 min, washing and addition of secondary antibody (anti-goat Alex 488, Life Technology) for further 30 min.

Flow cytometry was performed with BD FACSCanto^TMII^ or BD FACSLyric™ and data analysed with BD FACSDiva^TM^ Software or BD FACSuite™ Application (BD, Heidelberg, Germany). Using forward and sideward scatter single cells were gated. Living singlets were chosen from zombie-negative cells as described 31.

### Tissue staining

Tissue sections were stained by Masson trichrome staining. Immunofluorescence staining was performed with a S100A9 antibody (R&D) and anti-goat-Alexa546 (Invitrogen). Images were captured using KEYENCE BZ-9000 fluorescence microscope (Keyence GmbH, Neu-Isenburg, Germany).

### Cell isolation and culture

*Mouse myeloid cells* were generated from peritoneal lavage cells of C57BL/6 mice. Cells were cultured at 8 x 10^5^ cells/ml in RPMI 1640 medium (Thermo Fisher Scientific) containing 10% FCS (ThermoScientific), 1% penicillin/streptomycin (Biochrom GmbH), and 10 ng/ml M-CSF (Miltenyi) for 24 h. If indicated peritoneal-derived myeloid cells were stimulated as follows: 1.5 µg/ml recombinant murine S100A9 homodimer (R&D), 10 ng/ml TNFα (Miltenyi), or 10 ng/ml IL-1β (Miltenyi). In addition, cells were cultured in RPMI 1640 medium (Thermo Fisher Scientific) containing 5 mM glucose (Invitrogen), 10% FCS (ThermoScientific), 1% penicillin/streptomycin (Biochrom GmbH), and 5 ng/ml M-CSF (Miltenyi) for 24 h and then stimulated with 25 mM glucose. In co-stimulation experiments S100A9 homodimer was added 3 hours after addition of 25 mM glucose. For stimulation with FFA cells were incubated with 500 µM PA, SA or OA (Sigma-Aldrich) complexed to bovine serum albumin (BSA) as previously described 74. Cells treated with BSA were used as control. In co-stimulation experiments S100A9 homodimer was added 3 h after addition of FFA. In inhibition experiments 25 µg/ml Ac-YVAD-cmk (caspase-1 inhibitor), 5 µM Ca074 Me (cathepsin B inhibitor), 20 mM N-acetyl-L-cysteine or 10 µM diphenyliodoniumchloride (both ROS inhibitors, Sigma) were added together with S100A9 and 3 h after addition of FFA.

Differentiation of M1- and M2-like macrophages was induced in peritoneal-derived cells by addition of 100 ng/ml LPS (Sigma) / 20 ng/ml INFγ (Milteny) and 20 ng/ml IL-4 (Miltenyi) for 24 h, respectively. If indicated, S100A9 homodimer (1.5 µg/ml), S100A8 homodimer (1.5 µg/ml; R&D), or S100A8/A9 heterodimer (1.5 µg/ml; R&D) was added during M2-like differentiation. NFkB, MAPK, PI3K, TLR4 and RAGE signaling was inhibited by addition of 4 µM BMS-345541 (Hycultec GmbH, Beutelsbach, Germany), 10 nM Cobitinimib (Selleckchem, Houston, Texas, USA), 5 µM Apitolisib (Selleckchem), 1 µM CLI-095 (Invivogen) and 10 µM RAGE antagonistic peptide (R&D), respectively.

*Human peripheral blood monocytes* were isolated by magnetic bead isolation of CD14+ cells from blood of healthy volunteers in compliance with institutional ethical use protocols (number of the vote 428/16-ek). CD14+ monocytes were seeded in RPMI (Biochrom AG, Berlin, Germany) supplemented with 1% penicillin/streptomycin, 1% glutamine (Biochrom AG) and 10% heat inactivated FCS (Biochrom AG) at 0.5 × 10^6^ cells per well in the presence of 50 ng/ml M-CSF (Miltenyi).

*Keratinocytes*: A spontaneously immortalized mouse keratinocyte cell line was kindly provided by M. Magin (Institute of Biology and Translational Center for Regenerative Medicine, Leipzig University, 75. Cells were cultured in complete FAD Medium. FAD low Ca medium (Biochrom) was supplemented with 10% Chelex-treated FCS Gold (PAA, Pasching, Austria), 0. 18 mM adenine, 0.5 μg/ml hydrocortison, 5 μg/ml insulin, 100 pM choleratoxin (Sigma, St. Louis, MO), 10 ng/ml^-1^ EGF, 100 μg/ml penicillin/streptomycin, and 2 mM glutamax (Thermo Fisher Scientific), in 5% CO_2_ at 37°C 76. Stimulation was performed as described for myeloid cells. Human keratinocytes were prepared as described previously 77.

### *Ex vivo* skin culture

Mouse skin was cultured in supernatants from peritoneal cells after stimulation with FFA, S100A9 homodimer or co-stimulation with FFA and S100A9 homodimer and all controls as described above. For neutralization of IL-1β and TNFα activity 200 ng/ml IL-1 receptor antagonist (Invitrogen) or 1 µg/ml aTNFα (R&D) were added.

Mouse skin was stimulated *ex vivo* with 10 ng/ml IL-1β in the presence of NFkB inhibitor (BMS-345541, 4 µM, Hycultec GmbH) or MAPK inhibitor (Cobimitinib, 0.2 µM, Selleckchem). Skin samples were analyzed after 24 h of culture. Epidermis was separate by incubation with 0.25% trypsin (Sigma) for 1 h at 37°C. Dermal WAT was mechanically removed.

### Lipid extraction and analysis of free fatty acids

Serum aliquots (60 µl) were mixed with 1 ml methanol and centrifuged at 13,000 × g for 2 min to precipitate proteins. Lipids were extracted from the supernatant by the addition of 1 ml chloroform and water according to Bligh and Dyer 78. After vigorous shaking, samples were centrifuged for phase separation at 3,000 rpm for 5 min. The lower (organic) phase was withdrawn and lipid extraction was repeated by the addition of chloroform, vigorous shaking and centrifugation. Both organic phases of a sample were combined, evaporated to dryness and re-dissolved in 200 µl chloroform. Ten µl of the organic extracts were separated by high performance thin layer chromatography (HPTLC).The lipids in the FFA spot were automatically eluted by a Plate Express™ TLC plate reader (Advion, Ithaca, NY, USA) with methanol as solvent and directly analyzed by electrospray ionization mass spectrometry (ESI MS) as described 79. Total intensities of FFA and from these ratios for each FFA were calculated. Total FFA concentration was detected in serum samples by the Free Fatty Acid Quantitation Kit (Sigma-Aldrich; without lipid extraction) according to the manufacturer's protocol and detected via UV-spectrometer measurement (Hitachi U-2000; Hitachi Medical Systems America, Inc., Twinsburg, OH, USA) or Synergy HT (BioTek). Specific FFA concentrations were calculated from the percentage of specific FFA and total FFA.

### Statistics

Statistical analysis for two-group comparisons regarding normally distributed metrical data was performed using two-tailed Student's t test. Normality was tested by D'Agostino & Pearson Normality test or Shapiro-Wilk-Test (n≤4). Where normality was absent, Mann-Whitney test was used. For statistical comparison of more than two groups, ANOVA Test was used. As post-hoc test in case of significance of the ANOVA, Dunnet's T3 multiple comparison test was used. Calculations were done using GraphPad Prism version 7.02. P-values of 0.05 or smaller were consider statistically significant. The different degrees of significance were indicated as followed: * P < 0.05; ** P < 0.01; *** P < 0.001

### Study approval

All animal experiments were performed according to institutional and state guidelines and were approved by the Committee on Animal Welfare of Saxony (Germany, TVV65/13, TVV03/16, TVV13/19).

## Supplementary Material

Supplementary figures and table.
