# Supplementary material for: Overexpression of S100A9 in obesity impairs macrophage differentiation via TLR4-NFkB-signaling worsening inflammation and wound healing
Source: Theranostics. 2022 Jan 16;12(4):1659–82. doi: 10.7150/thno.67174 (PMC8825590; doi:10.7150/thno.67174)
Supplement: Supplementary file 1 — Supplementary figures and table. [file thnov12p1659s1.pdf]

Supplementary Materials

Supplemental Figure 1: Characterization of obese mice and short HFD mice

Supplemental Figure 2: Palmitic acid and high glucose do not alter M2-like macrophage differentiation.

Supplemental Figure 3: Validation of genome wide expression analysis

Supplemental Figure 4: TLR4-NFkB-axis mediates the dysregulation of M2-like macrophage differentiation by S100A9

Supplemental Figure 5: Inhibition of S100A9 does not affect early infiltration of inflammatory cells.

Supplemental Figure 6: S100A9 is not induced in fibroblasts, endothelial cells and tissue macrophages during inflammation

Supplemental Figure 7: S100A9 in the presence of saturated fatty acids stimulates IL-1β release from human monocytes

Supplemental Figure 8: Saturated fatty acids in combination with S100A9 induce IL-1β release from macrophages that in turn drives S100A9 overexpression in keratinocytes and dermal white adipose tissue

Supplemental Figure 9: Reduction of dietary saturated fatty acids restores M2-like macrophage differentiation and prevents exacerbation of skin inflammation in obesity.

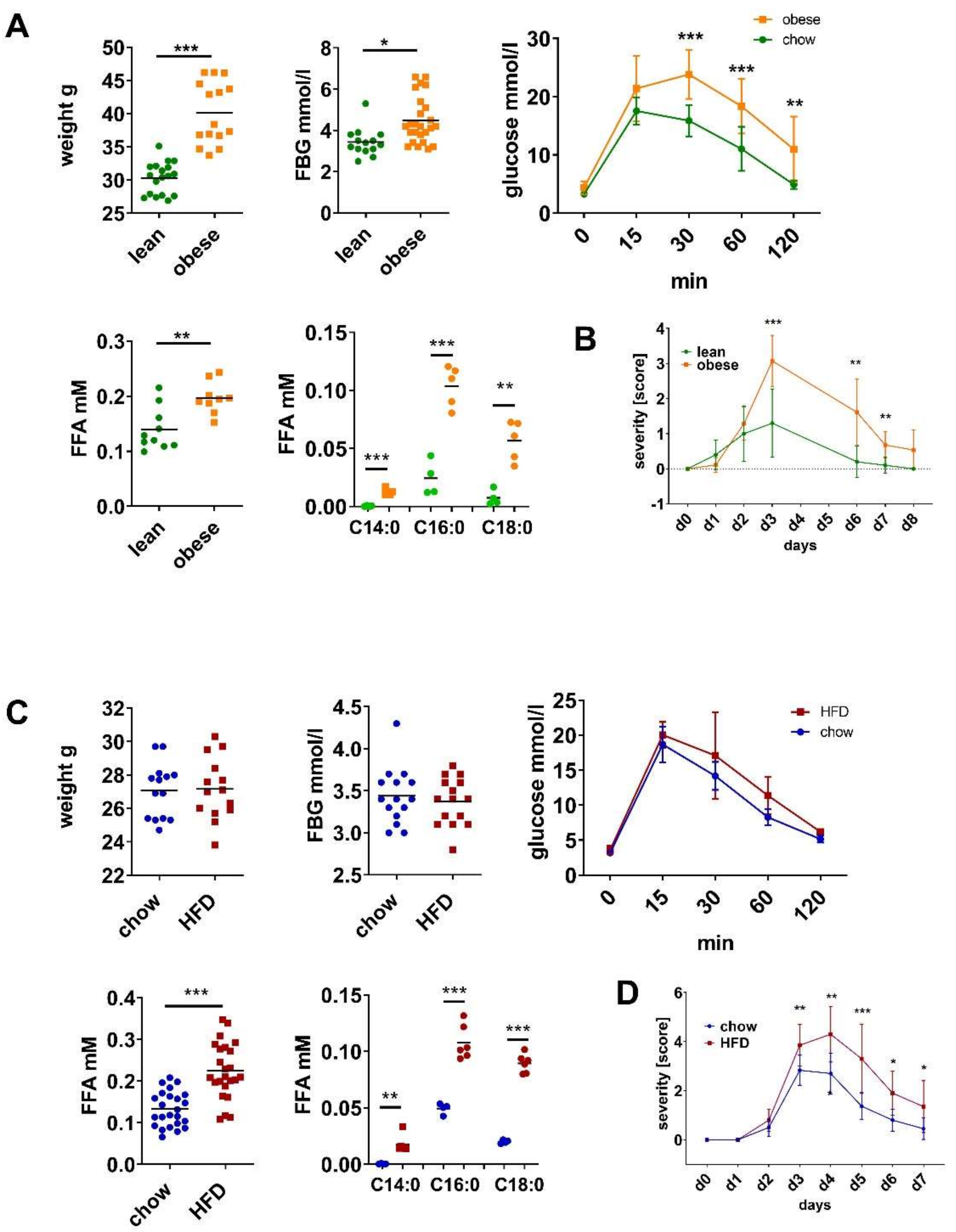

Supplemental Figure 1: Characterization of obese mice and short HFD mice. C57BL/6 mice were fed with high fat diet (HFD) or chow diet for **A/B)** 15 weeks (lean green; obese orange) and **C/D)** 6 weeks (chow blue; HFD red). **A/C)** Body weight, fasting blood glucose (FBG), glucose tolerance test and serum levels of free fatty acids (FFAs) were determined (C14:0 myristic acid; C16:0, palmitic acid; C18:0, stearic acid). **B/D)** Skin inflammation was induced by one topical application of imiquimod on the shaved back and severity of

inflammation was monitored. Each symbol represents one mouse. Mean is indicated. Unpaired t-test or Mann-Whitney : *P < 0.05, **P < 0.01, ***P < 0.001.

A

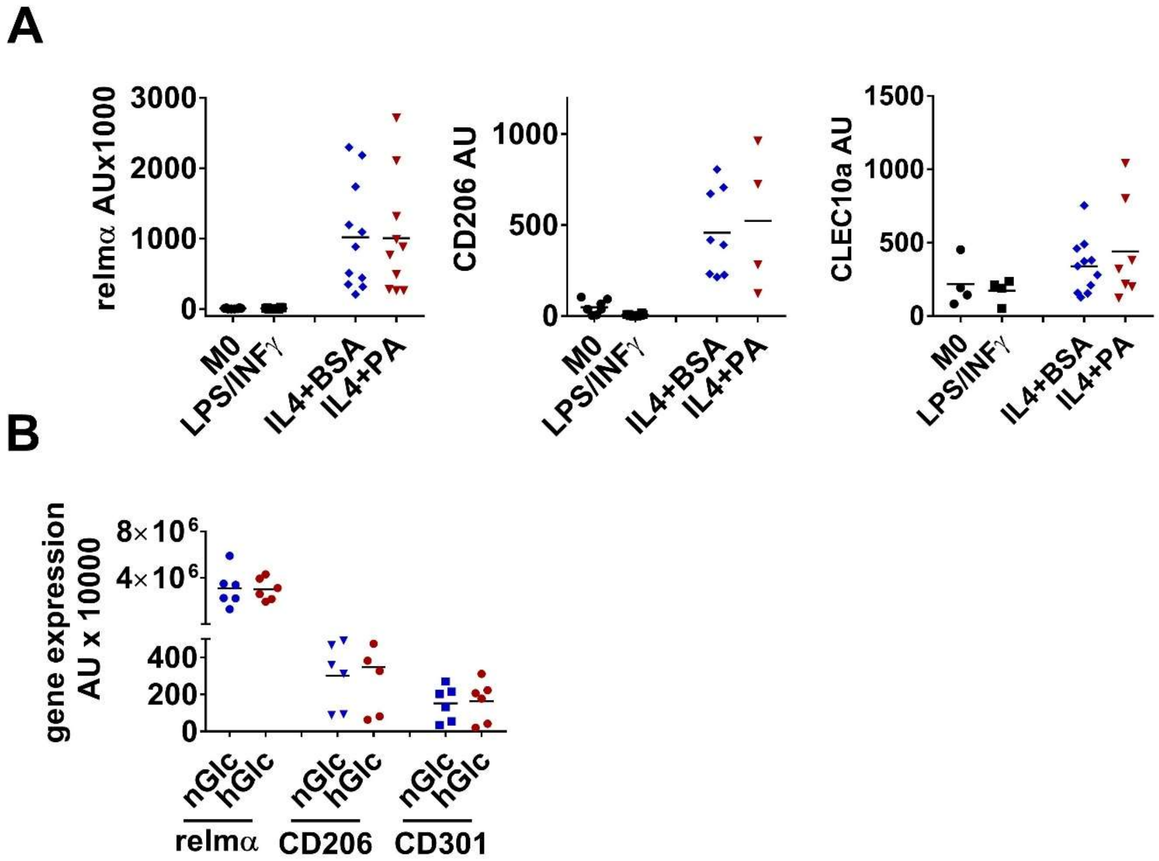

Supplemental Figure 2: Palmitic acid and high glucose do not alter M2-like macrophage differentiation. Myeloid cells were isolated from the peritoneum. M0-like macrophages were generated by culture with M-CSF while M1- and M2-like differentiation was induced by addition of LPS/INFγ (black) or IL-4 (blue, red), respectively. **A)** BSA (blue) or 500 µM BSA-palmitic acid (PA, red) complex were added during M2-like differentiation. **B)** Peritoneal cells were differentiated in the presence of 5 mM (blue, normal glucose, nGlc) or 25 mM (red, high glucose, hGlc) glucose. **A-B)** Relative expression of indicated genes normalized to RS36 detected by quantitative PCR. Each symbol represents one mouse. Mean is indicated as line.

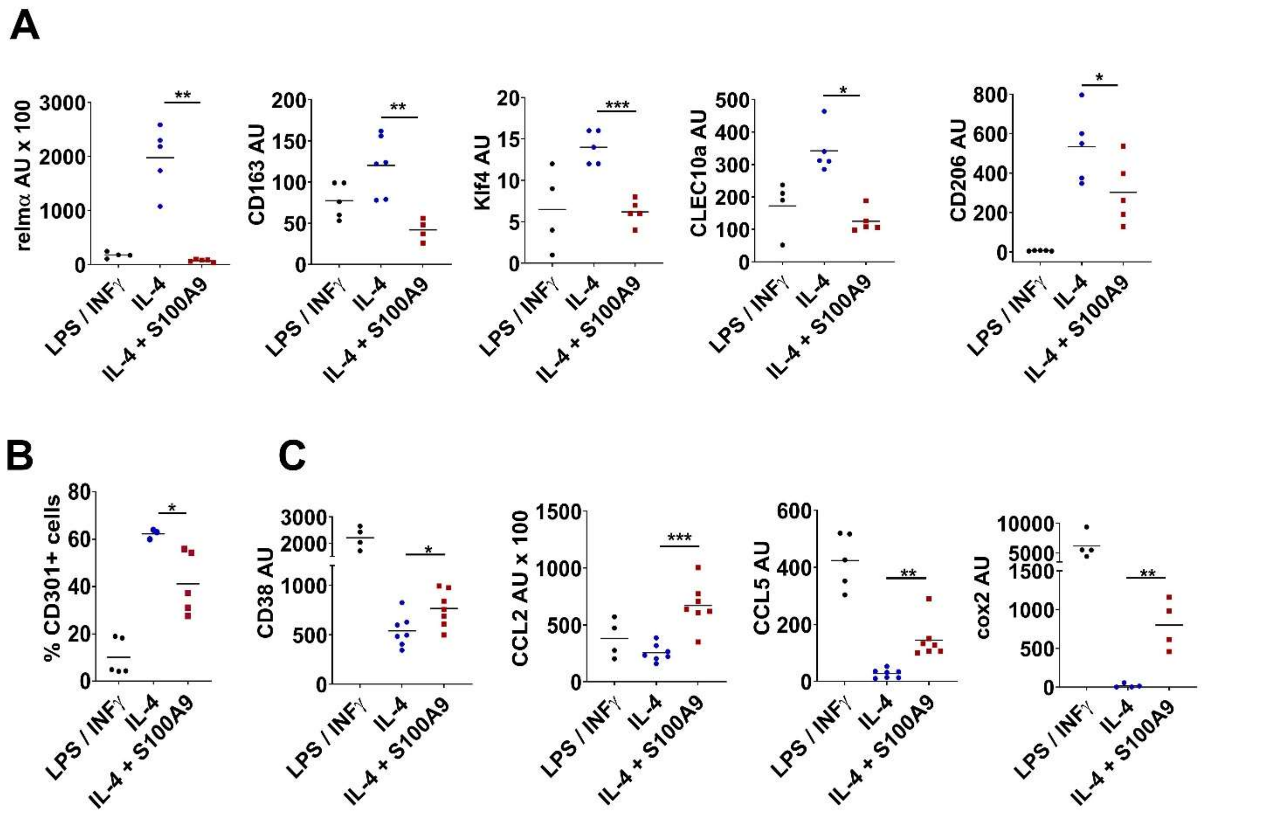

Supplemental Figure 3: Validation of genome wide expression analysis. Peritoneal macrophages were incubated with LPS / INFγ (black) or IL-4 (blue) to induce a M1 and M2-like macrophage phenotype, respectively. S100A9 was added during M2-like macrophage differentiation (red). **A)** Relative gene expression (RS36-normalized) of M2-marker genes detected by quantitative PCR. **B)** Quantification of CD301+ cells by flow cytometry. **C)** Relative gene expression (RS36-normalized) of M1-like genes by quantitative PCR. Each symbol represents macrophages from one mouse. Mean is indicated. Unpaired t-test or Mann-Whitney test between IL-4 and IL-4+S100A9-treated cells: *P < 0.05, **P < 0.01, ***P < 0.001.

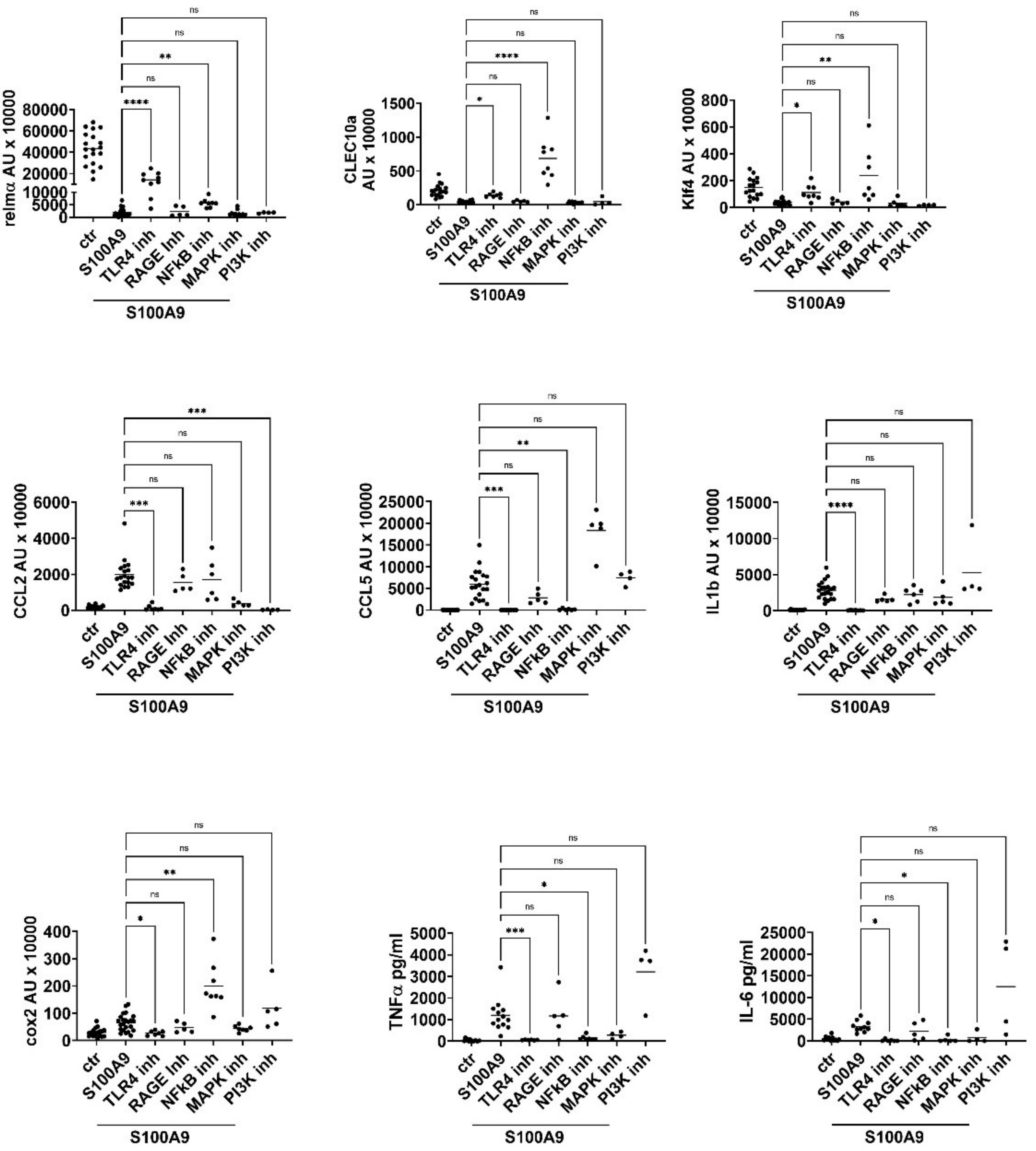

Supplemental Figure 4: TLR4-NFkB-axis mediates the dysregulation of M2-like macrophage differentiation by S100A9. Peritoneal macrophages were incubated with IL-4 (ctr) or IL-4 and S100A9 in the presence or absence of TLR4 signaling inhibitor (CLI-095, 1 µM), RAGE antagonistic peptide (ELKVLMEKEL, 10 µM), NFkB inhibitor (BMS-345541, 4 µM), PI3K inhibitor (Apitolisib, 5 µM), or MAPK-inhibitor (Cobimitinib, 10 nM). Gene expression was

detected by quantitative PCR normalized to RS36. The concentration of TNFα and IL-6 in the supernatant was determined by ELISA. Each symbol represents macrophages from one mouse. Mean is indicated. ANOVA with multiple comparisons between macrophages incubated with IL-4 and S100A9 in the presence of indicated inhibitors compared to macrophages incubated with IL-4 and S100A9. *$P < 0.05$, **$P < 0.01$, ***$P < 0.001$, ns – not significant.

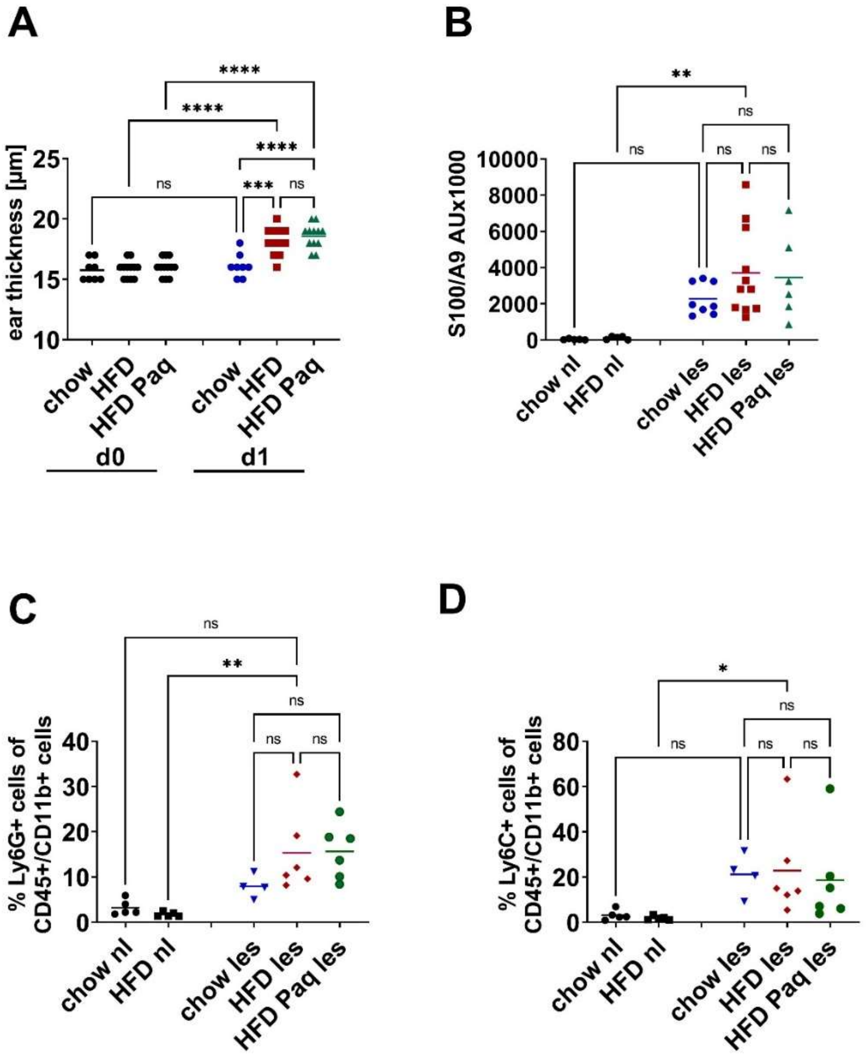

Supplemental Figure 5: Inhibition of S100A9 does not affect early infiltration of inflammatory cells. C57BL/6 mice were fed with chow diet (blue) or high fat diet (HFD, red) for 6 weeks. Skin inflammation was induced by one topical application of imiquimod on the shaved back and ears. After 10h administration of paquinomod was started (HFD Paq; green). Non-lesional (nl) and lesional skin (les) was analyzed after 16 h. **A)** Quantification of ear swelling. **B)** S100A9 expression normalized to RS36 detected by quantitative PCR. **C/D)** Quantification of infiltrating immune cells by flow cytometry. **C**) Relative amount of neutrophils (Ly6G+ cells among CD45+/CD11b+ cells); **D**) Relative amount of infiltrating monocytes (Ly6G-/Ly6C+ cells among CD45+/CD11b+ cells). Each symbol represents one mouse. Mean is indicated. ANOVA with multiple comparisons *P < 0.05, **P < 0.01, ***P < 0.001, ns – not significant.

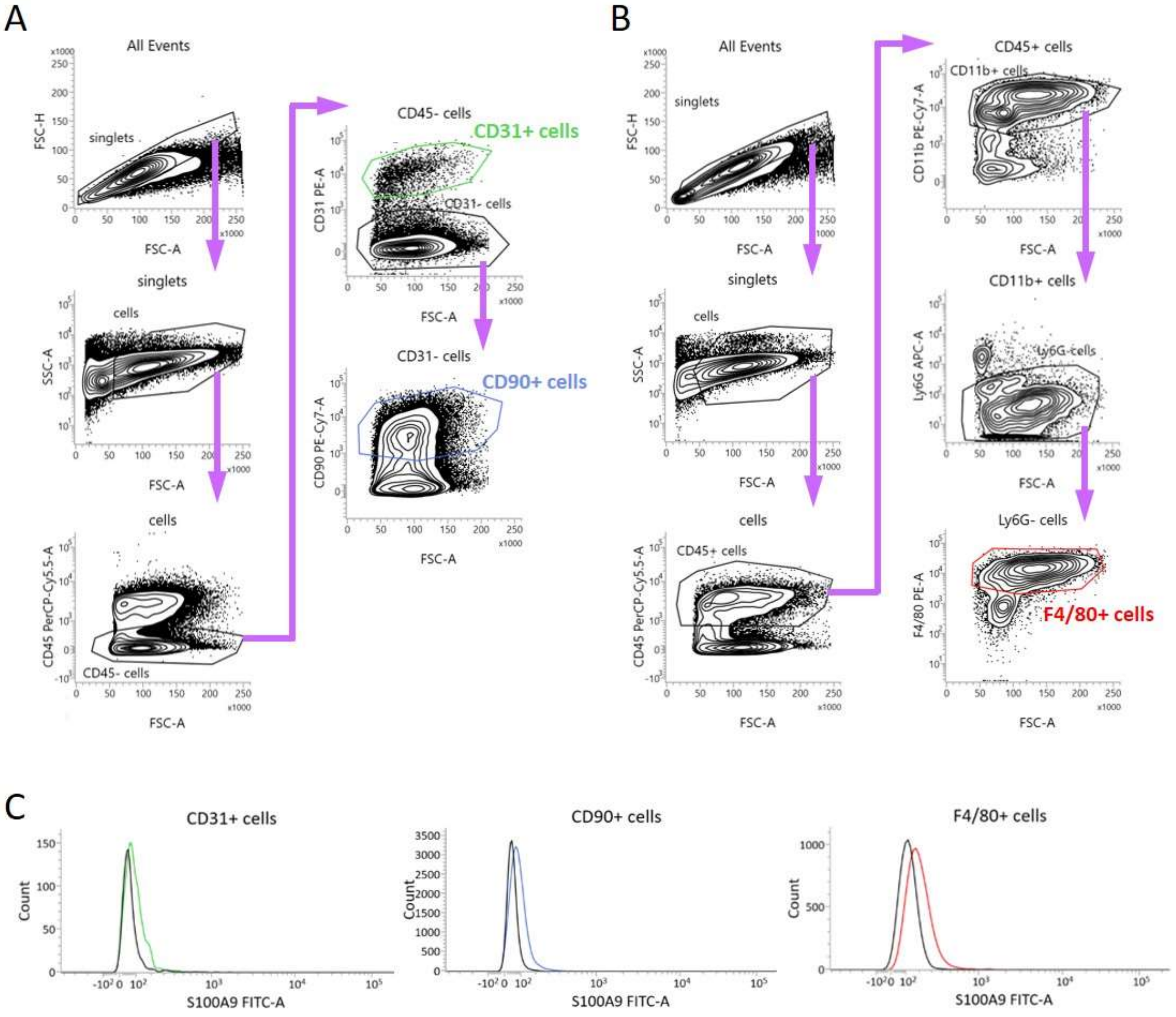

Supplemental Figure 6: S100A9 is barely expressed in fibroblasts, endothelial cells and macrophages during inflammation

Skin inflammation was induced by one topical application of imiquimod on the shaved back of C57BL6 mice. After 4 d S100A9 expression was detected in single cell suspension of lesional skin by flow cytometry. **A/B)** Gating strategies to define CD31+ endothelial cells and CD90+ fibroblasts **(A)**, and F4/80+ macrophages **(B)**. **C)** Detection of S100A9 in CD31+ endothelial cells, CD90+ fibroblasts, and F4/80+ macrophages. One representative histogram out of the analysis of skin cells from four mice.

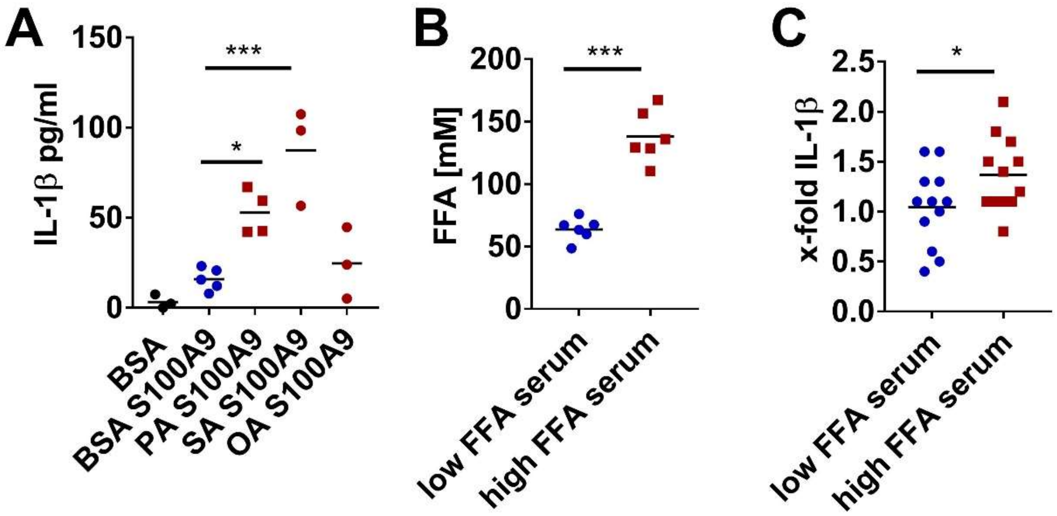

Supplemental Figure 7: S100A9 in the presence of saturated fatty acids stimulates IL-1β release from human monocytes. Human peripheral blood monocytes were isolated by magnetic bead isolation of CD14+ cells from blood of healthy volunteers. **A)** Stimulation with 500 µM palmitic acid (PA)-, stearic acid (SA)-, or oleic acid (OA)-BSA complexes or BSA alone followed by stimulation with S100A9. IL-1 β levels within the supernatants were detected by ELISA. **B)** Detection of total free fatty acids (FFA) in human serum used for stimulation of monocytes in C. **C)** Human CD14+ monocytes of two donors were incubated with serum containing low and high levels of FFA. IL-1ß concentration in the supernatant was determined after 48 h by ELISA. Each symbol represents one monocyte donor or serum donor. Mean is indicated as line. ANOVA with multiple comparisons (A) and unpaired t-test (B/C): *P< 0.05, **P< 0.01, ***P< 0.001.

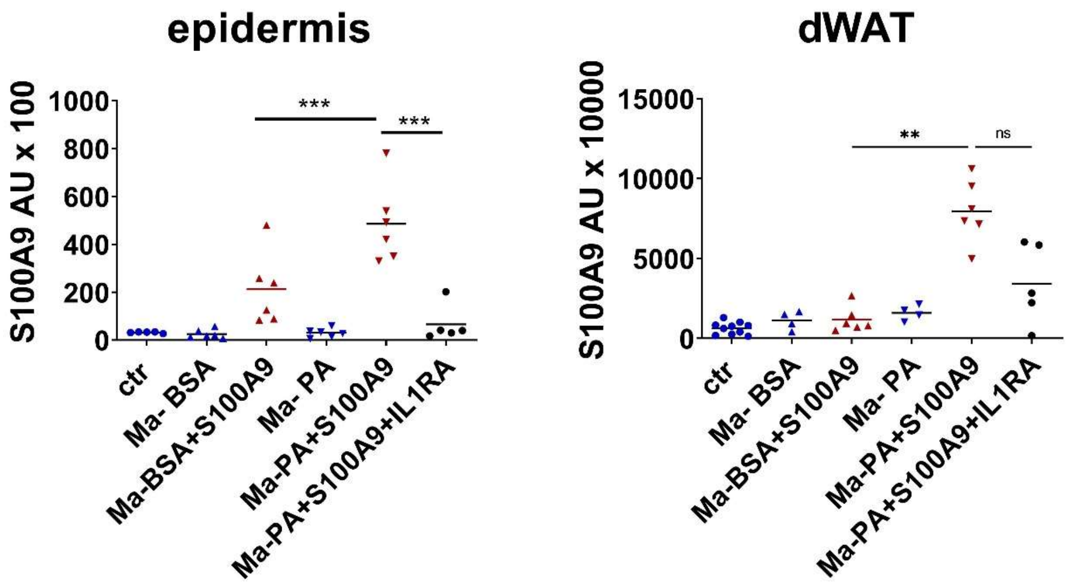

Supplemental Figure 8: Saturated fatty acids in combination with S100A9 induce IL-1β release from macrophages that in turn drives S100A9 overexpression in keratinocytes and dermal white adipose tissue (dWAT). Skin biopsies from lean chow-fed mice were incubated *ex vivo* with supernatants of PA-, BSA-, PA/S100A9- and BSA/S100A9-stimulated myeloid cells (Ma) or medium. IL-1β was neutralized by pre-incubation with 200 ng/ml IL-1 receptor antagonist (IL1RA). Relative expression of S100A9 (RS36-normalized) in the epidermis and dWAT separated from the skin after *ex vivo* culture was detected by quantitative PCR. Each dot represents cultured skin from one mouse. Mean is indicated. ANOVA with multiple comparisons: *P < 0.05, **P < 0.01, ***P < 0.001, ns – not significant.

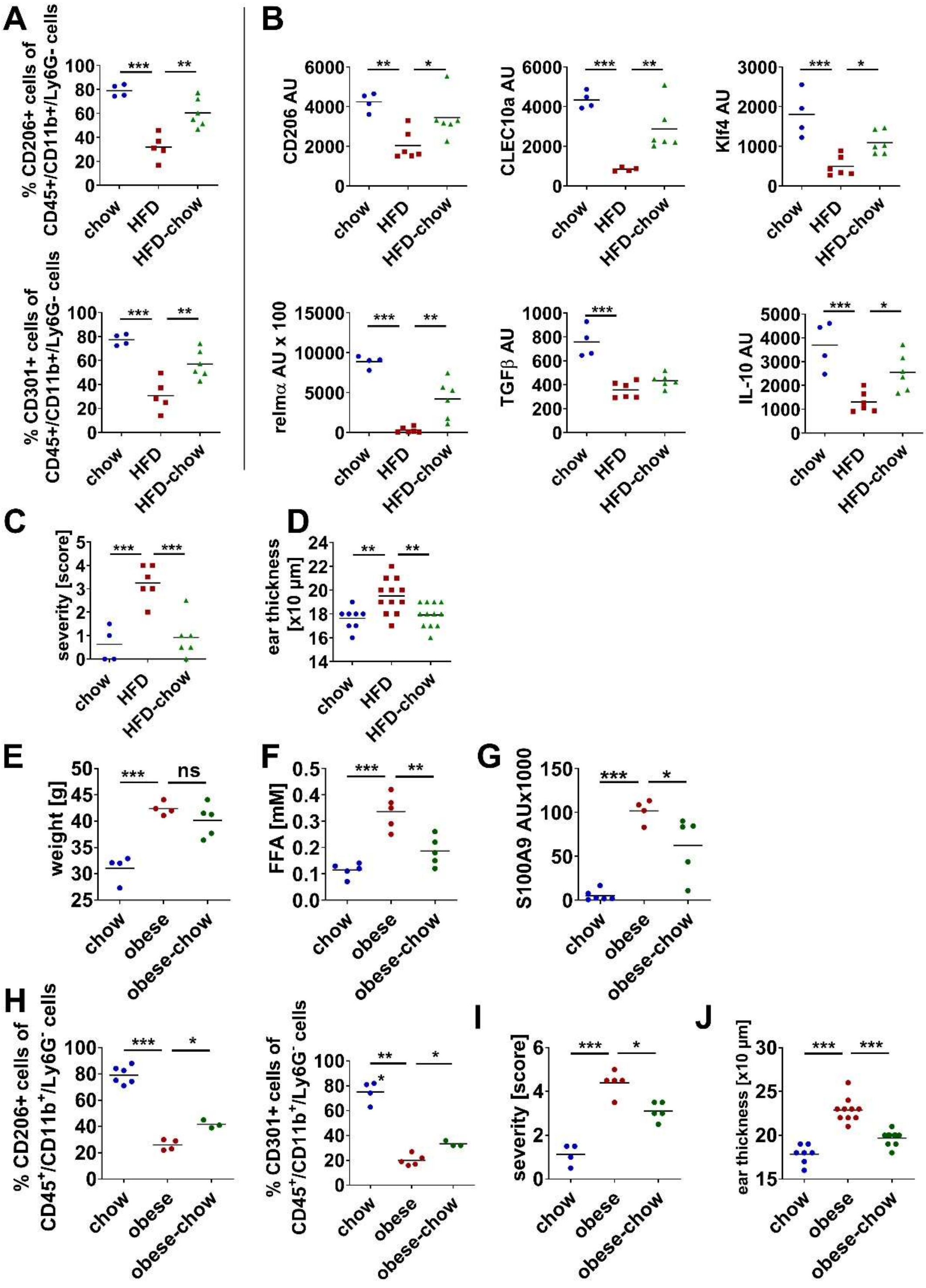

Supplemental Figure 9: Reduction of dietary saturated fatty acids restores M2-like macrophage differentiation and prevents exacerbation of skin inflammation in obesity. A-D) Male C57BL/6 mice received either high fat diet (HFD) for 6 weeks (red), HFD for 5 weeks followed by chow diet for one week (green), or chow diet for 6 weeks (blue). Skin inflammation was induced by one topical application of imiquimod on the shaved back and ears. Mice were

analyzed after 4d. **A)** Relative quantification of CD206+ and CD301+ macrophages within CD45+/CD11b+/Ly6G- macrophages in the lesional skin by flow cytometry. **B)** Relative expression of indicated genes (RS36-normalized) detected by quantitative PCR in CD11b+ myeloid cells isolated from lesional skin. **C)** Severity score, **D)** Ear swelling of both ears.

E-J) Male C57BL/6 mice received either HFD for 15 weeks (obese, red), HFD for 14 weeks followed by chow diet for one week (obese-chow, green), or chow diet for 15 weeks (chow, blue). Skin inflammation was induced by one topical application of imiquimod on the shaved back and ears. Mice were analyzed after 4 d. **E)** Body weight, **F)** Serum free fatty acids (FFA), **G)** Relative expression of S100A9 in the epidermis normalized to RS36 detected by quantitative PCR **H)** Quantification of CD206+ and CD301+ macrophages within CD45+/CD11b+/Ly6G- macrophages in the lesional skin by flow cytometry. **I)** Severity score. **J)** Ear swelling. Each symbol represents one mouse. Mean is indicated. ANOVA with multiple comparisons *P < 0.05, **P < 0.01, ***P < 0.001, ns – not significant.

.

Supplemental Table 1: Primer sequences

Gene (mouse)	Accession number	Sequence (5'-3')<br>Forward primer<br>Reverse primer
S100A9	NM_009114	ACTCTAGGAAGGAAGGACACCC<br>TACACTCCTCAAAGCTCAGCTG
IL-1β	NM_008361.3	GACAACTGCACTACAGGCTCC<br>AGGCCACAGGTATTTTGTCG
TNFα	NM_013693.3	CTTAGACTTTGCGGAGTCCG<br>ACAGTCCAGGTCACTGTCCC
CD38	NM_007646.5	CGCCTTGGTAGTAGGGATCG<br>ACAAGTGGGGCGTAGTCTTC
CCL2	NM_011333.3	TCAGCCAGATGCAGTTAACG<br>TCTGGACCCATTCCTTCTTG
Relmα	NM_020509.3	CCCTCCACTGTAACGAAGAC<br>CAACGAGTAAGCACAGGCAG
CLEC10A	NM_010796.3	CAGACCACTGGGCACAAAAC<br>TGGACGGAAACCAAGACACC
CD206	**NM_ 008625.2**	ACGGATAGATGGAGGGTGC<br>TGACTCTGGACACTTGCCAG
CD163	NM_001170395	CTGTGAGGGAAGAGTGGAGC

		TCCTCAGTTCCAGTGCAGTG
IL-10	NM_001160403.1	AGCCGGGAAGACAATAACTG<br>CATTTCCGATAAGGCTTGG
TGF β1	NM_011577	CTTCAATACGTCAGACATTCG<br>GTCAAAAGACAGCCACTCAG
Klf4	NM_010637.3	ATCAAGAGCTCATGCCACCG<br>CCTGTGTGTTTGCGGTAGTG
RS36	NM_007475	GGACCCGAGAAGACCTCCTT<br>GCACATCACTCAGAATTTCAATGG
